# Flemish Normative Data for the Buschke Selective Reminding Test

**DOI:** 10.5334/pb.486

**Published:** 2019-02-11

**Authors:** H. Thielen, G. Verleysen, S. Huybrechts, C. Lafosse, C. R. Gillebert

**Affiliations:** 1 Brain and Cognition, KU Leuven, Leuven, BE; 2 Faculty of Psychology and Educational Sciences, Ugent, Gent, BE; 3 Department Clinical Neuropsychology, RevArte Rehabilitation Hospital, Edegem, BE; 4 Department of Psychology, KU Leuven, Leuven, BE; 5 Department of Applied Psychology, Thomas More University College, BE

**Keywords:** verbal memory, selective reminding, Flemish normative data, age-associated memory impairment

## Abstract

The purpose of this study was to provide normative data for a Flemish version of the Buschke Selective Reminding Test (SRT). The SRT allows for the simultaneous analysis of several components of verbal memory, such as short and long term retrieval. The Flemish SRT was administered to 3257 neurologically healthy adults (1627 men and 1630 women, age range = 18–94 years). Effects of age, sex and education on SRT performance were assessed. Results indicate that SRT performance decreased with age and that this decline accelerated in men compared to women. Furthermore, an effect of education was found favoring participants who completed a higher education. Normative data quantified through percentile ranks and stratified by age, sex and education level are provided.

## Introduction

A well-functioning memory is essential in our everyday life. It allows us to remember who we are, what we did in the past and what we want to do in the near and far future. Furthermore, the memory processes of retention, recall and recognition are fundamental to learning. Unfortunately, these processes are susceptible to impairment in several neurological and psychiatric conditions (Baddeley, Kopelman, & Wilson, 2002). Even in neurotypical individuals, simply growing older can impair memory, which is known as age-associated memory impairment (Crook et al., 1986; McEntee, & Larrabee, 2000). Given its importance for everyday life and its vulnerability to ageing and disease, memory is a key element in neuropsychological assessment (Lezak et al., 2004). Consequently, several measures have been developed to assess different aspects of memory, including episodic memory, visuospatial memory, autobiographical memory and verbal memory (for a review, see Lezak et al., 2004 and Strauss, Sherman, & Spreen, 2006).

Verbal memory refers to the retention, recall and recognition of language-based material (Vanderploeg et al., 2001). The Auditory Verbal Learning Test (AVLT) has a history of regular use in the assessment of verbal memory (Boake, 2002; Lezak et al., 2004; Rey, 1958). The AVLT is a word-list learning test where a list of 15 unrelated items (list A) is read aloud during five consecutive trials. Immediate recall of this list follows each presentation. After the fifth trial, an interference list of 15 unrelated items (list B) is presented. Participants are then asked to recall list B prior to recalling list A. After a delay of 20 minutes, participants are asked once more to recall list A as well as to complete a recognition test. During the recognition test, participants have to distinguish items from list A from distractors (items from list B and items semantically or phonologically related to items from lists A or B). The AVLT is considered a conventional assessment of verbal memory due to the continuous presentation of items. The disadvantage of continuous presentation is that differentiation between retrieval from the short and long term memory is not possible (Lezak et al., 2004).

In contrast to the AVLT and other conventional verbal memory measures using continuous presentation, the Buschke selective reminding test (Buschke, 1973) uses selective presentation of the to be remembered items. The SRT is a word-list learning test where 12 unrelated items are presented during a maximum of 12 consecutive trials. Instead of presenting all the items simultaneously on each trial (i.e. continuous presentation), only the items that were not recalled on the previous trial are presented even though participants are instructed to recall the entire list of items on each trial.

Contrary to continuous presentation, selective presentation facilitates the distinction between short and long term retrieval. Since not all items are necessarily presented at the beginning of each trial, participants can recall items without being reminded of them at the beginning of the trial. While recall without reminding indicates retrieval from the long term memory, recall after reminding is assumed to tap onto short term memory.

The ability to differentiate between short and long term retrieval is beneficial for several reasons. For instance, an abnormally high dependence on short term memory during recall is indicative of an impairment in long term memory, which is a characteristic symptom of neurodegenerative disorders (Masur et al., 1989). Furthermore, short and long term retrieval can be impaired independently from one another (Della Sala et al., 2012). The selective presentation of items during the SRT allows for the simultaneous evaluation of several components of memory. In addition to short and long term retrieval, it assesses total recall, long term storage, multiple choice recognition, delayed recall and whether retrieval from the long term memory is organized or not (consistent long term retrieval).

The clinical value of the SRT is demonstrated by studies using the SRT to assess verbal memory function in patients with either acquired head injury (Leitner, Miller, & Libben, 2017), multiple sclerosis (Krupp, & Elkins, 2000), epilepsy (Bell et al., 2005), neurodegenerative and psychiatric disorders (including depression, schizophrenia and post-traumatic stress disorder) (Campo, Morales, & Martínez-Castillo, 2003; Goldberg et al., 1989; Semkovska, & McLoughlin, 2010; Vermetten et al., 2003). It is further supported by its ability to differentiate between different types of neurodegenerative disorders (Salmon et al., 2015) and its ability to predict progression from age-associated cognition to mild cognitive impairment (Blacker et al., 2007).

Because of its widespread use, different versions of the SRT have been developed. These include four parallel English versions, a Greek, a Spanish, and a Hebrew version (Campo, & Morales, 2004; Gigi et al., 1999; Hannay, & Levin, 1985; Larrabee et al., 1988; Zalonis et al., 2009). Normative data demonstrate that age, sex and education significantly influence SRT performance (Campo, & Morales, 2004; Gigi et al., 1999; Larrabee et al., 1988; Wiederholt et al., 1993; Zalonis et al., 2009). Generally, memory declines with age, women outperform men and higher education is associated with better SRT performance. In addition, performance of men declines more rapidly with increasing age than performance of women and performance of individuals with lower education declines more rapidly compared to individuals that completed a college education (Wiederholt et al., 1993). Similar effects of age, sex and education were found for other verbal memory measures including the AVLT (e.g. Miatton et al., 2004).

The decrease in verbal memory performance with increasing age can be explained by the higher incidence of mild cognitive impairment and dementia in older adults (aged over 60) (e.g. Lobo et al., 2000; Yesavage et al., 2002), even though non-pathological ageing also results in worse verbal memory (Harada, Natelseon Love, & Triebel, 2013). Other explanations offered for age-associated memory decline include genetic contributions (for a review see Small, 2001), structural and functional brain changes (e.g. Cabeza et al., 1997; Cabeza et al., 2000; Pelletier et al., 2017), decreased health status (i.e. higher incidence of cardiovascular disease at older age) (Rafnsson et al., 2007) and lifestyle changes (i.e. limited physical activity at older age) (Arrieta et al., 2018) (for a review see Deary et al., 2009). For verbal memory specifically, age-related structural and functional brain changes include atrophy in the medial temporal lobe (Pelletier et al., 2017) and decreased right prefrontal activity and increased left prefrontal activity during retrieval in older adults when compared to younger adults (Cabeza et al., 1997; Cabeza et al., 2000). Lastly, age-related decline in other cognitive functions such as processing speed and executive functioning (e.g. strategy use) also influence memory performance (Davis et al., 2013; Salthouse, 2000).

Similarly, various explanations are offered regarding sex differences in verbal memory performance. Neurobiological explanations include differences in hormonal influences, brain anatomy and physiology (Chen et al., 2007; Filipek et al., 1994; Sherwin, 2003) (for a review see Andreano, & Cahill, 2009). Structural brain differences have also been suggested to explain the accelerated decline of memory performance in men compared to women. Gur and colleagues (1991) found increased and faster brain atrophy in older men compared to older women (aged 55 and up). Furthermore, for men atrophy was dominant in the left hemisphere while for women atrophy was more symmetrical in both hemispheres. Furthermore, sex differences have been associated with superior encoding in women due to usage of more efficient encoding strategies than men (Guillem, & Mograss, 2005; Krueger, & Salthouse, 2010). Lastly, social explanations have been offered including difference in sociodemographic variables and health habits between men and women (Jorm et al., 2004).

Results on the effect of education on SRT performance are inconsistent, but generally higher education is related to better performance (Campo, & Morales, 2004; Larrabee et al., 1988; Zalonis et al., 2009). This effect can be linked to the cognitive reserve theory. In the cognitive reserve theory, the brain is thought to use certain processes to actively cope with brain pathology in order to compensate for possible cognitive impairments (Stern, 2002; Stern, 2009). One of the processes supporting this coping mechanism is education. Other factors include socioeconomic status, intelligence, occupational attainment and mental stimulation which are associated with education (Stern, 2009).

Since a Flemish SRT was currently lacking, the English SRT (version 2) was translated in Flemish using back-translation by independent translators (Verleysen, 2012). The objectives of the current study are to provide normative data for the Flemish SRT, considering different demographic characteristics. This will allow for the investigation of the effects of age, sex and education on verbal memory performance. Based on previous studies (Campo, & Morales, 2004; Gigi et al., 1999; Wiederholt et al., 1993; Zalonis et al., 2009), we expect a negative association between verbal memory and age, we expect women to perform better than men and we expect higher education to be associated with better SRT performance.

## Method

### Participants

We recruited 3257 neurologically healthy volunteers (1627 men and 1630 women). First year bachelor students Applied Psychology at the Thomas More University College in Antwerp helped recruit participants. To earn extra credits on their final examination, students each had to recruit two to three participants. Exclusion criteria for participants were a history of neurological, cardiovascular or psychiatric disease. All participants reported that Flemish was their dominant language.

The total sample was aged 18 to 94 years (*M* = 45.32; *SD* = 18.53). The data were stratified according to six age categories ranging from 18 to 29 years (n = 825), 30 to 39 years (n = 459), 40 to 49 years (n = 521), 50 to 59 years (n = 622), 60 to 69 years (n = 389) and all ages above 70 (n = 441). There was no significant difference in age between men (*M* = 45.16 years, *SD* = 18.47, range = 18–94 years) and women (*M* = 45.47, *SD* = 18.59, range = 18–92 years) (*t*(3255) = –0.48; *p* = 0.63). Further stratification per age category considered four educational levels: level 1 included primary and elementary education (maximum 12 years of education, no secondary school diploma), level 2 included a secondary school diploma with the emphasis on preparation for specific jobs (technical secondary education and vocational secondary education), level 3 included a general secondary school diploma and a maximum of three years of higher education, level 4 included every education with minimum four years of higher or university education (master’s degree or higher). The distribution of participants across the different demographic variables is presented in Table 1.

**Table 1:** Distribution of participants across age category, sex and education level.

Sex	Education Level	Age (in years)						Total
		18-29	30-39	40-49	50-59	60-69	70+	

Men	1	105	44	61	56	44	54	364
	2	131	74	64	79	58	51	457
	3	117	57	59	92	35	52	412
	4	53	64	75	93	50	59	394
Total		406	239	259	320	187	216	1627

Women	1	62	28	66	63	52	61	332
	2	134	60	69	66	55	58	442
	3	148	58	68	80	46	54	454
	4	75	74	59	93	49	52	402
Total		419	220	262	302	202	225	1630

Education level 1: primary and elementary education. Education level 2: technical secondary education and vocational secondary education. Education level 3: general secondary school diploma and bachelor degree. Education level 4: master’s degree or higher.

### Materials and procedure

The second version of the English SRT (Hannay, & Levin, 1985) was translated to Flemish (Verleysen, 2012) (see Table 2). The items of the newly formed list were words that frequently occur in the Flemish language, are normally acquired in Flemish speaking individuals with primary education, and had no apparent semantic or phonetic association with the other items in the list. The amount of syllable nouns per item ranged from one to three. From the original list 11 items were retained, the item “disagree” was changed to meet above criteria. Since there is no suitable Flemish word for “disagree”, it was changed to “agree”. The Flemish SRT, including the Flemish instructions, are included in Appendix A.

**Table 2:** The original second version of the English version of the SRT and the adapted Flemish list.

Original list	Adapted list	Flemish translation

Shine	Shine	Schijn
Disagree	Agree	Akkoord
Fat	Fat	Dik
Wealthy	Wealthy	Rijk
Drunk	Drunk	Dronken
Pin	Pin	Pin
Grass	Grass	Gras
Moon	Moon	Maan
Prepare	Prepare	Bereiden
Prize	Prize	Prijs
Duck	Duck	Eend
Leaf	Leaf	Blad

Data were collected in a quiet room without distraction. Participants were asked to give verbal informed consent prior to the data acquisition. Subsequently, the examiner asked the participants (1) information on basic demographic information (age, sex and education level); (2) if they had a probable history of neurological, psychiatric or cardiovascular disease; and (3) if Flemish was their primary language. If participants reported a history of neurological, cardiovascular or psychiatric disease or limited knowledge of the Flemish language acquisition, the SRT was not administered and participants were excluded from the study.

The SRT was administered following the procedure described by Buschke (1973). The examiner read each item aloud at a rate of one item per two seconds. The participant had to recall as many items in any possible order. Afterwards, the examiner only presented the items that the participant had not recalled on the immediately preceding trial. Again, the participant had to try to recall as many items as possible from the entire list of 12 items. This procedure was repeated for 12 trials or until the participant recalled the entire list of 12 items on three consecutive trials without needing any reminding. The learning trials were followed by a multiple choice recognition task. Subjects were given four items which included an item from the list, a semantically related item, a phonemic related item and an unrelated item. From this list participants had to select the item that was present in the learned list. This was repeated for each item of the list. After a delay of 30 minutes, the participant had to again recall as many items as possible without receiving any reminders concerning the original list.

Scoring of the test performance again followed the procedure of Buschke (1973; Buschke, & Fuld, 1974). The test allows for simultaneous examination of several measures: total recall, long term retrieval, long term storage, short term retrieval, consistent long term retrieval, number of correct recognized multiple choice items and delayed recall. The meaning of the different measures is explained in Table 3. For each learning trial, the number of intrusions was also recorded. The total recall, long term retrieval, short term retrieval, long term storage and consistent long term scores were calculated by adding the scores of the twelve individual trials. If the test was concluded prematurely because the participants recalled all the items on three consecutive trials a maximum score was given for the following trials.

**Table 3:** Definitions of the different verbal memory measures of the SRT.

Measure	Definition

Total recall	The total amount of items recalled.
Long term storage	Items recalled on two successive trials without intermediate reminding enter the long term storage on the first of these two trials. These items belong to the long term storage on all consecutive trials regardless of whether the items were recalled or not.
Long term retrieval	Items recalled that belong to the long term storage and thus are recalled without reminding.
Short term retrieval	Items recalled that do not belong to the long term storage and thus are recalled after reminding.
Consistent long term retrieval	Items that have entered the long term storage and are recalled without intermediate reminding on at least two trials. This indicates organized retrieval from the long term memory.
Multiple choice recall	The number of correctly recognized items during the multiple choice recognition task.
Delayed recall	The number of recalled items during the delayed recall.

### Data analysis

**Descriptive statistics.** Since the dependent variables (total recall, long term retrieval, long term storage, short term retrieval, consistent long term retrieval, multiple choice recognition and delayed recall) did not follow a normal distribution normative data were quantified through percentile ranks. The normative data were stratified according to sex, age category and education level (see Appendix B). The normative data for total recall are displayed in Table 4 for males and in Table 5 for females.

**Table 4:** Normative data (percentile ranks) of males for total recall, stratified according to age category and education level.

Education level	Percentile	Age (in years)					
		18-29	30-39	40-49	50-59	60-69	70+

1	1	66.12	47.00	58.00	58.00	42.00	34.00
	2	68.36	47.00	61.12	58.42	42.00	34.30
	5	86.50	79.75	77.10	69.50	46.75	39.25
	10	97.20	88.00	80.20	77.00	53.50	54.50
	25	109.00	94.50	95.00	86.00	73.25	60.75
	50	120.00	113.00	110.00	96.50	86.50	76.50
	75	127.00	122.00	119.50	107.25	100.75	93.75
	90	134.00	127.50	129.80	124.30	120.50	118.00
	95	134.70	130.75	132.00	126.20	136.50	130.25
	97	136.64	132.30	133.14	133.29	137.65	132.40
	99	140.88	133.00	134.00	134.00	138.00	135.00

2	1	70.64	45.00	71.00	65.00	49.00	41.00
	2	90.92	57.50	71.60	73.40	49.90	41.28
	5	95.00	81.75	79.50	81.00	55.90	48.60
	10	99.20	89.00	84.50	83.00	61.60	56.80
	25	113.00	102.00	94.25	95.00	81.00	73.00
	50	123.00	116.00	109.00	103.00	95.00	85.00
	75	131.00	128.25	118.75	119.00	109.25	101.00
	90	136.80	135.00	132.00	128.00	117.00	116.80
	95	137.00	137.00	134.00	130.00	120.25	121.20
	97	139.00	137.75	136.00	132.60	126.61	124.32
	99	142.36	140.00	136.00	138.00	132.00	126.00

3	1	88.36	81.00	88.00	65.00	21.00	43.00
	2	91.44	83.88	88.00	70.16	21.00	43.54
	5	98.00	99.00	89.00	81.25	53.00	52.65
	10	109.80	104.80	92.00	88.00	70.20	63.30
	25	117.50	112.00	101.00	101.00	78.00	75.25
	50	128.00	125.00	118.00	109.50	104.00	91.50
	75	134.00	131.50	129.00	122.00	119.00	104.00
	90	137.20	135.20	134.00	131.00	127.00	114.40
	95	139.10	137.30	136.00	135.70	137.40	122.05
	97	140.46	140.26	138.80	138.21	138.84	126.05
	99	143.00	141.00	142.00	143.00	139.00	129.00

4	1	104.00	89.00	72.00	57.00	58.00	30.00
	2	104.00	89.30	78.76	70.20	58.16	30.20
	5	104.70	97.75	92.20	75.80	68.75	50.00
	10	118.00	105.50	97.00	89.20	81.30	58.00
	25	123.50	116.00	109.00	106.00	91.75	81.00
	50	131.00	125.50	125.00	119.00	105.50	92.00
	75	136.00	133.00	130.00	128.50	115.50	122.00
	90	140.00	137.00	135.40	134.00	132.20	129.00
	95	142.00	139.75	137.20	136.30	135.00	132.00
	97	142.38	141.05	138.72	138.18	135.94	138.40
	99	143.00	142.00	140.00	143.00	137.00	140.00

Education level 1: primary and elementary education. Education level 2: technical secondary education and vocational secondary education. Education level 3: general secondary school diploma and bachelor degree. Education level 4: master’s degree or higher.

**Table 5:** Normative data (percentile ranks) of females for total recall, stratified according to age category and education level.

Education level	Percentile	Age (in years)					
		18-29	30-39	40-49	50-59	60-69	70+

1	1	76.00	73.00	71.00	52.00	57.00	37.00
	2	76.52	73.00	75.08	56.48	57.06	38.44
	5	84.45	77.05	92.35	75.40	58.65	56.00
	10	95.60	92.80	97.00	79.20	71.20	60.20
	25	105.75	111.00	104.00	89.00	84.00	72.50
	50	121.00	115.50	116.00	106.00	101.00	89.00
	75	130.25	125.75	127.00	119.00	113.00	102.50
	90	135.00	130.20	131.30	128.40	127.20	122.80
	95	138.00	132.55	133.65	132.60	130.35	130.80
	97	140.11	133.00	137.96	134.00	131.00	132.70
	99	141.00	133.00	138.00	134.00	131.00	137.00

2	1	84.10	91.00	63.00	54.00	52.00	43.00
	2	88.00	91.88	70.60	60.46	52.60	43.18
	5	101.75	95.20	91.00	86.10	61.00	49.70
	10	108.00	102.10	98.00	95.80	68.60	54.70
	25	117.00	108.25	110.00	106.75	76.00	70.50
	50	127.00	121.00	122.00	119.00	100.00	90.50
	75	134.00	130.00	128.50	128.00	118.00	103.00
	90	138.00	133.00	135.00	134.00	125.00	118.30
	95	140.00	133.00	135.50	136.65	126.20	123.05
	97	141.00	137.02	136.90	138.98	128.28	124.69
	99	142.00	142.00	138.00	142.00	131.00	127.00

3	1	99.94	80.00	87.00	94.00	69.00	48.00
	2	103.00	82.34	88.14	94.00	69.00	49.60
	5	114.45	103.45	94.35	101.00	72.35	67.00
	10	117.90	110.90	106.00	103.00	82.20	73.00
	25	124.00	115.00	116.00	113.00	96.00	87.50
	50	131.00	125.50	124.50	123.50	106.00	100.00
	75	135.00	136.00	132.00	129.00	119.25	117.50
	90	139.00	139.00	135.00	134.80	126.50	128.00
	95	140.00	140.00	136.00	136.00	132.95	133.25
	97	141.00	140.46	136.93	137.00	136.95	134.70
	99	142.53	142.00	140.00	138.00	139.00	136.00

4	1	94.00	96.00	98.00	97.00	68.00	40.00
	2	97.64	96.50	99.20	97.88	68.00	40.24
	5	112.60	100.25	104.00	102.00	76.00	51.80
	10	121.60	113.00	110.00	109.40	85.00	63.30
	25	128.00	123.00	119.00	119.50	102.00	84.25
	50	134.00	130.00	127.00	128.00	114.00	108.50
	75	137.00	134.25	134.00	134.00	127.50	120.00
	90	140.40	137.50	139.00	137.00	135.00	128.40
	95	142.00	141.50	142.00	139.00	135.50	134.35
	97	142.00	143.00	142.40	139.36	136.50	137.05
	99	143.00	144.00	144.00	142.00	137.00	140.00

Education level 1: primary and elementary education. Education level 2: technical secondary education and vocational secondary education. Education level 3: general secondary school diploma and bachelor degree. Education level 4: master’s degree or higher.

**Figure 1:**
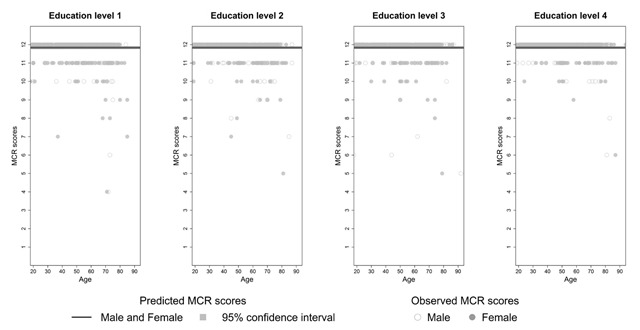
Predicted and observed multiple choice recognition scores (MCR) stratified according to sex and education level.

**Effects of demographic variables on SRT performance.** Due to the presence of outliers, heterogeneity of variances and non-normal distribution of residuals each of the dependent variables (except multiple choice recognition) was submitted to a robust regression (Field, & Wilcox, 2017; Maronna, & Yohai, 2000; Susanti et al., 2014). The variables sex and education level were dummy coded using men as a reference group for sex and level 1 for education level. Age was added to the regression model as a continuous variable. Since the relationship between age and SRT performance was non-linear, a polynomial regression including a quadratic relationship between age and SRT performance was computed. Interactions were only added to the model if they significantly increased the goodness-of-fit (with α = 0.05). The robust regression procedure determined outliers for each dependent variable and gave them a weight of zero without having to delete the outliers. There were two outliers for total recall, one outlier for long term retrieval, 20 outliers for short term retrieval, 17 for long term storage and 102 for delayed recall. Since multiple choice recognition reached a ceiling effect in most participants (see Figure 1) a negative binomial regression including the same independent variables was proposed. However, when comparing this model to an intercept only model it proved that the latter fit the data equally well (χ^2^ (16) = 7.29, *p* = 0.97). Other studies reported a similar lack of variability in multiple choice recognition performance (Campo, & Morales, 2004; Larrabee et al., 1988). All statistical analyses were performed using R 3.5.1. (R core Team, 2013).

## Results

### Descriptive statistics

Means and standard deviations for all the dependent measures of the SRT stratified by sex, age category and education level can be found in Tables C1 and C2 (see Appendix C).

### Effects of demographic variables on SRT performance

The results discussed below are confined to the significant effects of age, sex and education on SRT performance. All statistics are presented in Appendix C (see Tables C3–C8). The predictors included in the robust regression model explained a significant amount of variance in the different dependent variables (see Table 6).

**Table 6:** Proportion of variance explained and overall significance of the robust regression model for each dependent variable.

Dependent variable	Adjusted *R*^2^	df_1_	df_2_	*F* statistic	*P*

TR	0.43	10	3246	1793.3	*p* < 0.01**
LTR	0.41	10	3246	1652	*p* < 0.01**
STR	0.30	10	3246	972	*p* < 0.01**
LTS	0.38	10	3246	1260.3	*p* < 0.01**
CLTR	0.37	8	3248	1762.8	*p* < 0.01**
DR	0.38	16	3240	719.7	*p* < 0.01**

TR = total recall; LTR = long term retrieval; STR = short term retrieval; LTS = long term storage; CLTR = consistent long term retrieval; DR = delayed recall.

**Effects of age on SRT performance.** For each dependent variable, the relationship between age and SRT performance was quadratic (see Appendix C, Tables C3–C8). For consistent long term retrieval and delayed recall there was also a significant linear relationship between age and SRT performance (see Appendix C, Tables C7 and C8). The robust regressions revealed that, when controlling for effects of sex and educational level, total recall performance decreased with increasing age (β_age*age_ = –0.01, *t*(3246) = –9.06, *p* < 0.01; β_age*sex_ = 0.14, *t*(3246) = 3.90, *p* < 0.01; β_age*level 4_ = 0.20, *t*(3246) = 3.41, *p* < 0.01). Similar results were found for long term retrieval, long term storage, consistent long term retrieval and delayed recall (see Appendix C, Tables C4 and C6–C8). From previous studies regarding the SRT (Campo, & Morales, 2004; Zalonis et al., 2009) we learned that with increasing age individuals start to rely more on short term retrieval and less on long term retrieval. Therefore, we expected a positive relationship between age and short term retrieval. We found that, when controlling for effects of age and educational level, short term retrieval increased with increasing age (β_age*age_ = 0.0007, *t*(3246) = 5.83, *p* < 0.01; β_age*sex_ = –0.05, *t*(3246) = –2.62, *p* < 0.01; β_age*level 4_ = –0.10, *t*(3246) = –3.58, *p* < 0.01)

**Figure 2:**
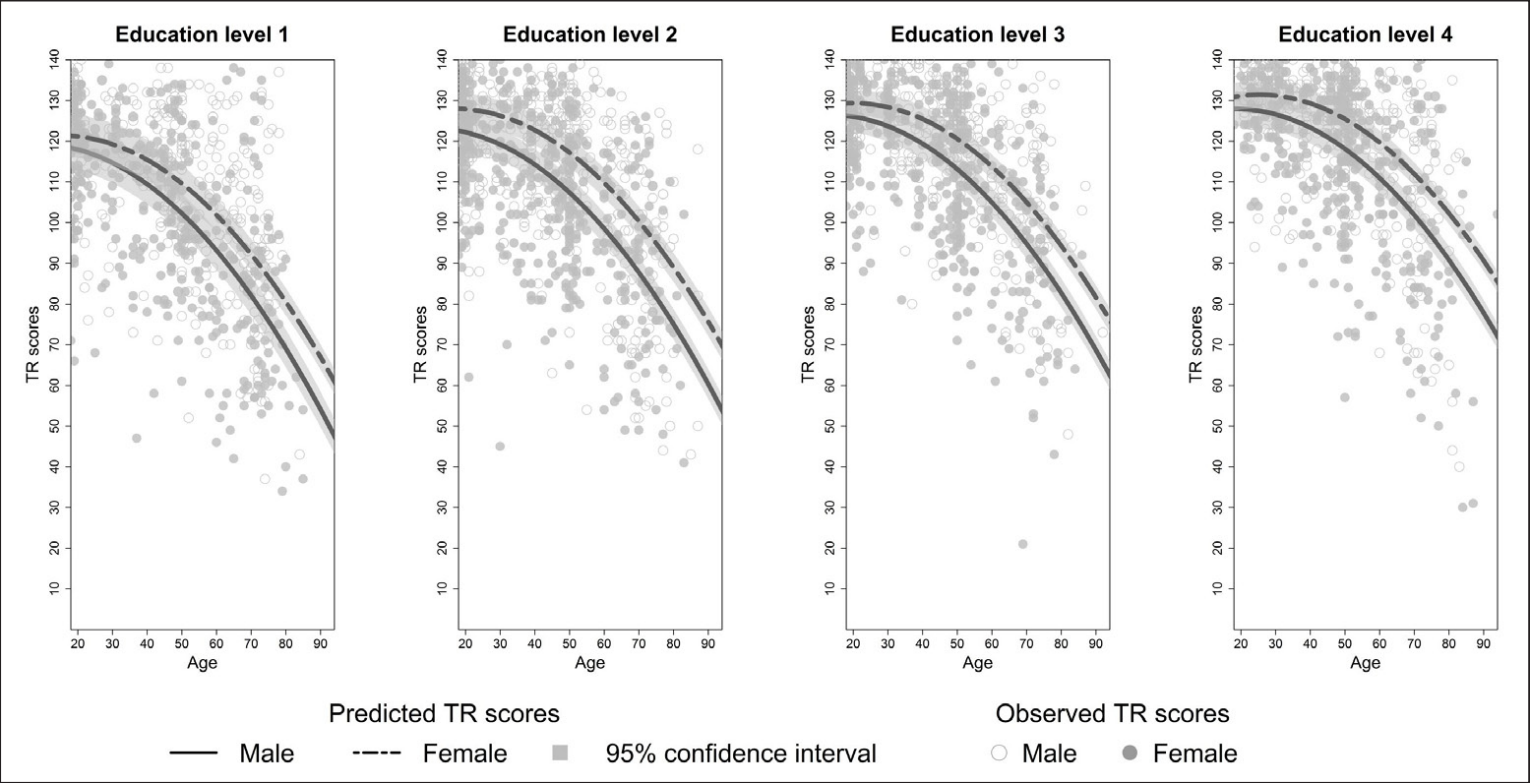
Predicted and observed total recall scores (TR) stratified according to sex and education level.

**Figure 3:**
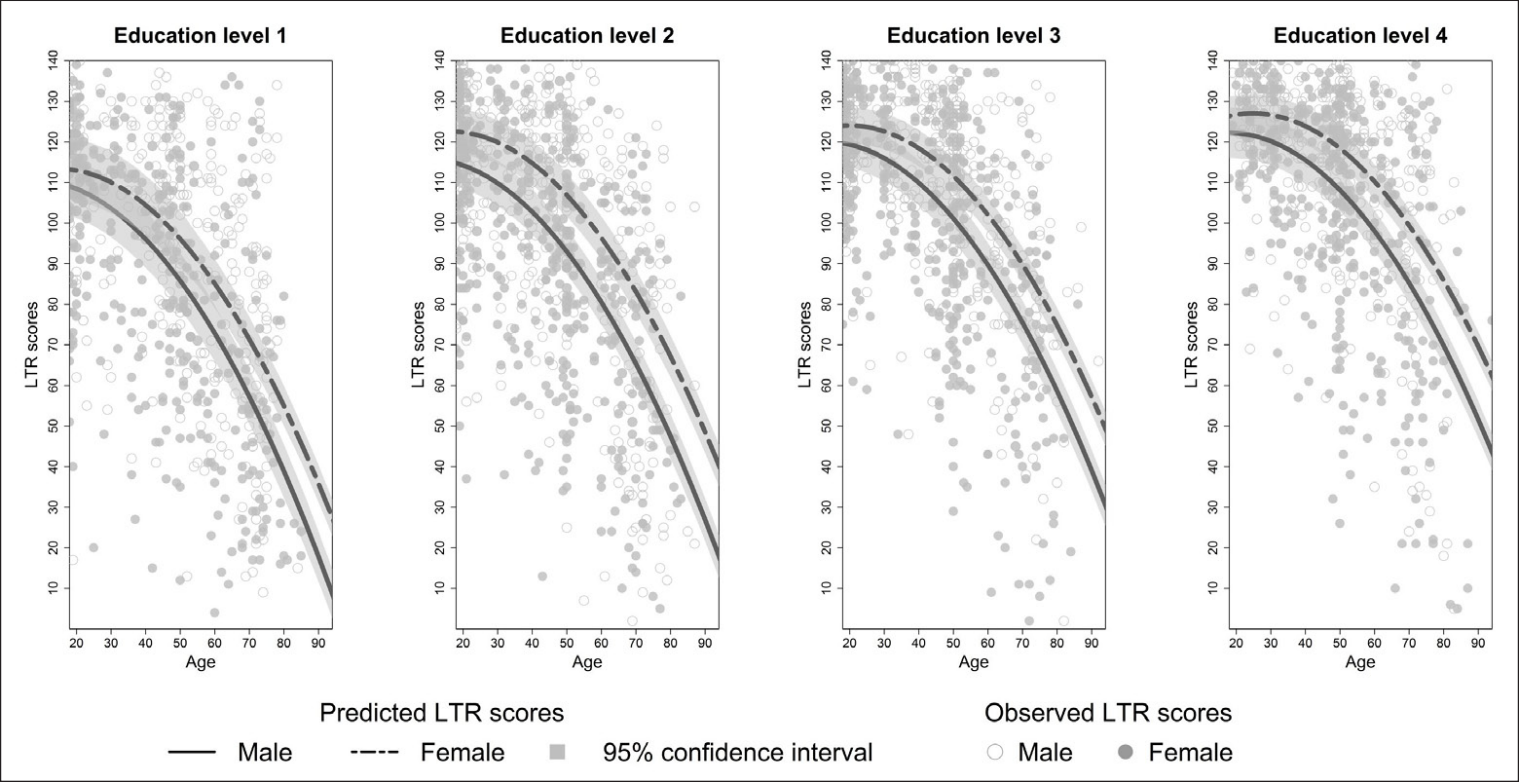
Predicted and observed long term retrieval scores (LTR) stratified according to sex and education level.

**Figure 4:**
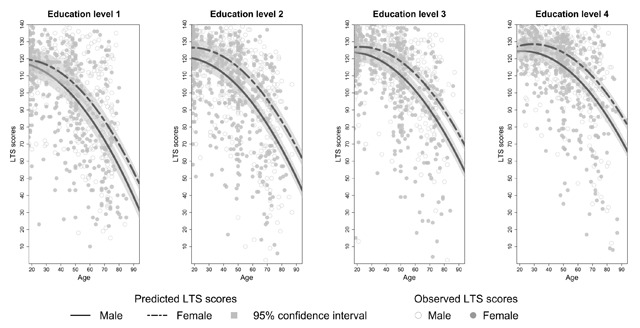
Predicted and observed term storage scores (LTS) stratified according to sex and education level.

**Figure 5:**
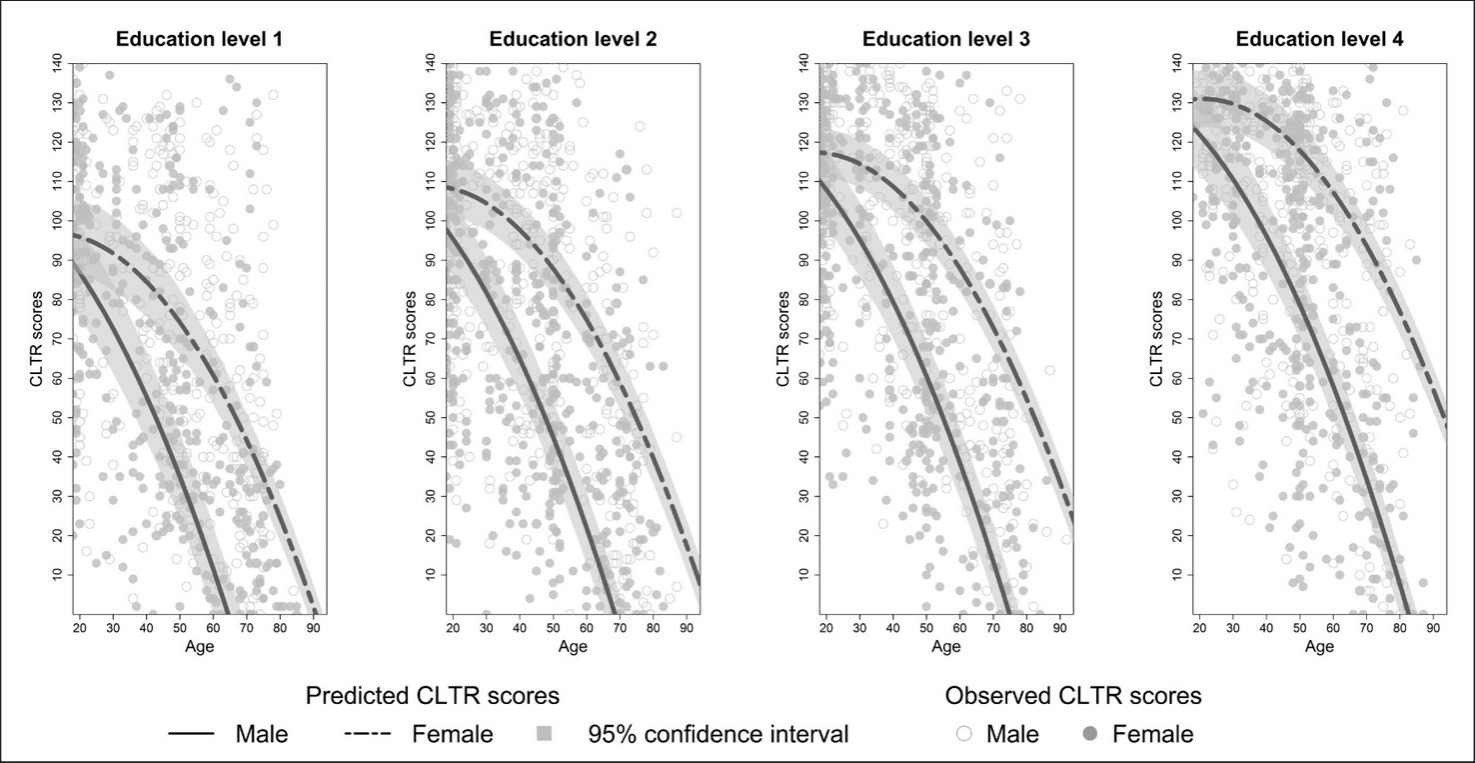
Predicted and observed consistent long term retrieval scores (CLTR) stratified according to sex and education level.

**Figure 6:**
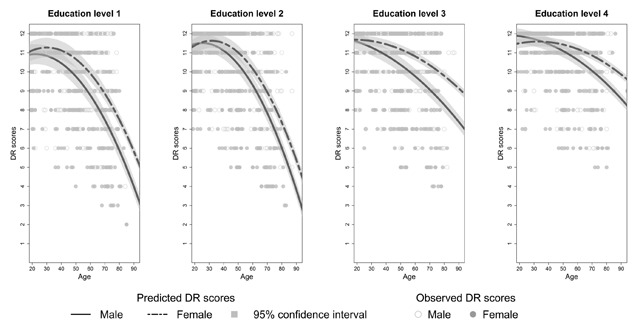
Predicted and observed delayed recall scores (DR) stratified according to sex and education level.

**Figure 7:**
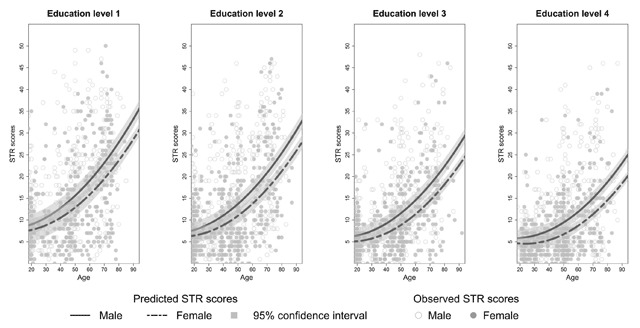
Predicted and observed short term retrieval scores (STR) stratified according to sex and education level.

**Effects of sex on SRT performance.** There was no main effect of sex on SRT performance but sex interacted significantly with age for each dependent variable (see Appendix C, Tables C3–C8). The decline in total recall with increasing age differed significantly between the two sexes (β_age*sex_ = 0.14, *t*(3246) = 3.90, *p* < 0.01). Similar results were found for long term retrieval, long term storage, consistent long term retrieval and delayed recall (see Appendix C, Tables C4 and C6–C8). From Figures 2 to 6 it seems that a faster decline in SRT performance is visible in men compared to women. The higher dependence on short term retrieval with increasing age also differed significantly between the two sexes with accelerated retrieval from the short term memory in men compared to women (β_age*sex_ = –0.05, *t*(3246) = –2.62, *p* < 0.01) (see Figure 7).

**Effects of education on SRT performance.** Total recall performance was better for education level 3 and 4 compared to level 1 (*β_level 3_* = 6.23, *t*(3246) = 3.06, *p* < 0.01; *β_level 4_* = 6.02, *t*(3246) = 2.50, *p* = 0.01). Similar effects were seen for long term retrieval (*β_level 3_* = 7.97, *t*(3246) = 2.69, *p* < 0.01; *β_level 4_* = 7.99, *t*(3246) = 2.29, *p* = 0.02) and for consistent long term retrieval when comparing education level 2, 3 and 4 to level 1 (*β_level 2_* = 7.65, *t*(3248) = 4.31, *p* < 0.01; *β_level 3_* = 18.17, *t*(3248) = 10.41, *p* < 0.01; *β_level 4_* = 29.35, *t*(3248) = 16.24, *p* < 0.01). Additionally, for total recall education level interacted significantly with age but this interaction was limited to education level 4 (β_age*level 4_ = 0.20, *t*(3246) = 3.41, *p* < 0.01). For long term retrieval, long term storage and delayed recall this interaction was significant for both education level 3 and 4 (see Appendix C, Tables C4, C6 and C8). For these variables, the decline in SRT performance with increasing age seems more gradual for education level 3 and 4 compared to education level 1 (see Figures 2, 3, 4 and 6). Regarding short term retrieval, it seems that the higher dependence on short term retrieval with increasing age was less apparent when comparing education level 4 to level 1 (β_age*level 4_ = –0.10, *t*(3246) = –3.58, *p* < 0.01) (see Figure 7). Lastly, for delayed recall the interaction between education level and sex was significant but only when regarding education level 4 (β_sex*level 4_ = –0.57, *t*(3240) = –2.56, *p* = 0.01). The sex difference in delayed recall seems to be smaller regarding education level 4 when compared to level 1 (see Figure 6).

## Discussion

The aim of this study was to provide normative data for a Flemish version of the Buschke Selective Reminding Test and assess influences of age, sex and education on SRT performance. Our hypotheses were that SRT performance would decrease with age, women would outperform men and that receiving a higher education would increase SRT performance.

Several limitations of this study should be mentioned. Firstly, the age group 18 till 29 years old was disproportionally large. Since students were responsible for participant recruitment it is not surprising that this was the easiest age category to recruit. Secondly, memory performance of the age group 70 years and older was very heterogeneous. It would be interesting to divide this age group in smaller categories (e.g. categories of 5 years). The small number of participants older than 80 prevented us to do this in the current study. In future research it is advised to include a screening for neurodegenerative disorders (e.g. the Montreal Cognitive Assessment) (Nasreddine et al., 2005) to be able to divide the older cohorts into adults with mild cognitive impairment, dementia or neurotypical memory impairment. Lastly, due to the availability of parallel forms of the SRT (Hannay, & Levin, 1985) it would be interesting to translate the parallel versions to Flemish to allow for repeated administration of the SRT.

Consistent with previous research (Campo, & Morales, 2004; Gigi et al., 1999; Wiederholt et al., 1993; Zalonis et al., 2009), SRT performance decreased with increasing age. Since only neurologically healthy volunteers were included this decline is thought to reflect age-associated memory impairment (McEntee, & Larrabee, 2000). From Figures 2 to 7 it seems that after a certain age the decline in SRT performance starts to accelerate. Furthermore, this point of acceleration seems to differ according to sex and educational level. Longitudinal research is needed to confirm these findings.

Regarding sex differences in verbal memory performance, the results do not point to a boosted performance in women compared to men irrespective of their age but to sex differences in the age-associated memory decay. The inconsistency with previous studies reporting a significant main effect of sex is possibly due to these studies omitting the interaction between age and sex in their statistical model and to a substantial difference in sample size (Campo, & Morales, 2004; Gigi et al., 1999; Zalonis et al., 2009). For future research examining the effect of sex on verbal memory performance it is encouraged to contemplate an interaction between age and sex in addition to main effects of sex.

Our results suggest that age-associated memory decline is more gradual in women than in men. Because of the cross-sectional design of this study differences between the different age groups have to be interpreted cautiously. Therefore, longitudinal research is needed to confirm our results. Furthermore, future research is advised to investigate the presumed multifactorial origin of sex differences in memory decline. Current explanations for this sex difference range from neurobiological explanations such as more and faster left hemisphere atrophy in men (Gur et al., 1991), to neuropsychological differences such as the usage of more efficient encoding strategies in women (Guillem, & Mograss, 2005; Krueger, & Salthouse, 2010) and social differences such as differences in health habits (Jorm et al., 2004).

Regarding the association between education and SRT performance, we found a significant effect of education level on SRT performance. The inconsistency with a previous study reporting no influence of education on SRT performance is possibly due to a significantly larger sample size in the current study (Larrabee et al., 1988). Similar to Campo and Morales (2004) and Wiederholt et al. (1993) our results illustrate the importance of attending higher education for verbal memory performance. Higher education had a positive effect on SRT performance and interacted with age to predict SRT performance. Specifically, similar to Wiederholt et al. (1993) our results suggest that the age-associated decay is more gradual in participants who attended higher versus lower education. This can be linked to the cognitive reserve theory (Stern, 2002; Stern, 2009) where education is considered an active process that can help compensate for cognitive impairments. Again, longitudinal research is needed to confirm these findings.

In conclusion, this study provided normative data in healthy Flemish adults. The data pointed to influences of age, sex and education level on SRT performance. Therefore, normative data was stratified according to these variables.

## Data Accessibility Statement

The datasets acquired and analyzed during the current study and an electronic scoring aid that compares observed scores to predicted scores are available on figshare at [https://doi.org/10.6084/m9.figshare.7471124 and https://doi.org/10.6084/m9.figshare.7471130.v1 respectively] and can be acquired from the corresponding author [H. Thielen] on request. Additionally, the dataset was donated to the Advanced Neuropsychological Diagnostics Infrastructure project (de Vent et al., 2016).

## Additional Files

The additional files for this article can be found as follows:

10.5334/pb.486.s1 Appendix A. Flemish version of the SRT.

10.5334/pb.486.s1 Appendix B. Normative data for the Flemish SRT.

10.5334/pb.486.s1 Appendix C. Supplementary tables.
