# Supplementary material for: Flemish Normative Data for the Buschke Selective Reminding Test
Source: Psychol Belg. 2019 Feb 11;59(1):58–77. doi: 10.5334/pb.486 (PMC6625541; doi:10.5334/pb.486)
Supplement: Appendix A. — Flemish version of the SRT. [file pb-59-1-486-s1.pdf]

Appendix A. Flemish version of the SRT.

Instructie 1: reproductie van de 12 woorden

Voor het aanbieden van de eerste testbeurt:

"Deze geheugentest bestaat uit twaalf worden die ik langzaam zal voorlezen. Luister aandachtig en tracht zoveel mogelijk woorden te onthouden. Nadat ik het laatste woord heb uitgesproken, zal ik u namelijk vragen zoveel mogelijk van die woorden op te sommen. In welke volgorde u dat doet is niet van belang. Niet alle mensen zijn in staat zich alle woorden in één keer te herinneren. Daarom zal ik telkens de woorden nog eens zeggen die u vergat op te noemen.

We gaan dit een aantal keren herhalen. Belangrijk is wel dat het de bedoeling is dat u steeds zoveel mogelijk- en dus niet enkel de laatst vernoemde – woorden opsomt. Heb is dan ook vooral belangrijk dat u gewoon zo goed mogelijk uw best doet.

Heeft u nog vragen?"

Na het aanbieden van eerste testbeurt:

"Zo, dit waren alle woorden. Welke woorden weet u nog?"

Voor het aanbieden van de tweede testbeurt:

"Wat ik nu ga doen, is enkel dié woorden voorlezen die u zonet vergat op te noemen. U zal nadien worden gevraagd niet alleen deze 'nieuwe' woorden, maar ook de door u zonet wel vernoemde woorden opnieuw op te sommen. Ook nu heeft de volgorde uiteraard geen belang."

Voor het aanbieden van de verdere testbeurten:

"De volgende woorden was u vergeten: …. . Probeer ze nu terug allemaal op te noemen."

Instructie 2: multiple choice herkenning

Voor het aanbieden van de multiple choice herkenning:

"Ik ga u nu telkens vier woorden voorlezen. Keer op keer zit er een woord tussen dat u zonet al eerder heeft gehoord tijdens het voorlezen van de twaalf woorden. U zegt mij telkens welk woord het was."

Instructie 3: uitgestelde herinnering

Voor het aanbieden van de uitgestelde herinnering (na 30 minuten):

"Ik ga u nu nog een laatste keer vragen zoveel mogelijk van die woorden van de geheugentest op te sommen. In welke volgorde u dat doet is hierbij niet van belang. Welke woorden weet u nog?"

	1	2	3	4	5	6	7	8	9	10	11	12		DR
1. schijn														
2. akkoord														
3. dik														
4. rijk														
5. dronken														
6. pin														
7. gras														
8. maan														
9. bereiden														
10. prijs														
11. eend														
12. blad														
													Totaal:	
TR														
LTR														
STR														
LTS														
CLTR														
Intrusies														
MCR														
DR														

Multiple choice herkenning (het juiste woord is onderlijnd):

1. schijn – glans – klein – bank
2. gelijk – omtrek – verhoor – akkoord
3. dik – vet – boom – pik
4. dans – geld – rijk – slijk
5. pronken – dronken – zat – hevig
6. kin – pen – plank – pin
7. glas – gras – plan – wei
8. maan – ster – nacht – baan
9. beroven – klaarmaken – bereiden – verhuizen
10. winst – prijs – pot – spijs
11. schors – gans – eend – eind
12. klad – stoel – vel - blad

Appendix B. Normative data for the Flemish SRT.

Table B1

Normative data (percentile ranks) for men aged 18-29, stratified according to education level

Education level	Percentile	TR	LTR	STR	LTS	CLTR	DR	MCR
1 (n = 105)	1	66.12	21.20	0.06	24.62	13.12	4.06	10.00
	2	68.36	40.96	1.12	50.84	15.60	5.00	10.00
	5	86.50	66.00	2.00	70.50	29.90	7.00	11.00
	10	97.20	72.60	3.00	81.00	46.60	8.00	12.00
	25	109.00	94.00	5.00	102.50	67.50	9.00	12.00
	50	120.00	110.00	9.00	117.00	99.00	11.00	12.00
	75	127.00	121.50	15.00	125.50	114.50	12.00	12.00
	90	134.00	130.00	21.40	131.00	127.00	12.00	12.00
	95	134.70	131.70	26.70	135.00	130.00	12.00	12.00
	97	136.64	134.82	32.74	136.82	134.28	12.00	12.00
	99	140.88	138.88	47.34	138.94	138.88	12.00	12.00
2 (n = 131)	1	70.64	41.16	0.00	53.24	18.32	5.32	10.32
	2	90.92	59.60	0.00	65.68	24.12	6.00	11.00
	5	95.00	74.40	1.00	85.40	39.80	7.60	12.00
	10	99.20	84.20	2.00	95.20	49.20	9.00	12.00
	25	113.00	103.00	4.00	110.00	68.00	11.00	12.00
	50	123.00	116.00	7.00	121.00	100.00	12.00	12.00
	75	131.00	126.00	12.00	129.00	121.00	12.00	12.00
	90	136.80	134.00	19.00	135.00	132.80	12.00	12.00
	95	137.00	137.00	23.00	137.40	136.40	12.00	12.00
	97	139.00	137.04	25.04	138.04	137.04	12.00	12.00
	99	142.36	141.36	35.80	141.36	141.36	12.00	12.00
3 (n = 117)	1	88.36	59.36	0.00	24.36	33.36	6.00	11.18
	2	91.44	66.04	0.00	68.44	35.00	6.36	12.00
	5	98.00	80.70	0.00	87.90	53.90	8.00	12.00
	10	109.80	96.00	1.00	100.80	68.00	9.00	12.00
	25	117.50	107.00	3.00	113.50	87.00	11.00	12.00
	50	128.00	122.00	5.00	126.00	113.00	12.00	12.00
	75	134.00	132.00	10.50	133.00	129.00	12.00	12.00
	90	137.20	135.20	15.00	138.00	134.20	12.00	12.00
	95	139.10	138.00	19.20	139.10	138.00	12.00	12.00
	97	140.46	140.46	25.22	141.46	140.46	12.00	12.00
	99	143.00	142.82	32.64	143.00	142.82	12.00	12.00
4 (n = 53)	1	104.00	83.00	0.00	89.00	43.00	7.00	10.00
	2	104.00	83.08	0.00	89.32	43.64	7.00	10.16
	5	104.70	90.30	0.00	101.40	53.80	7.70	12.00
	10	118.00	109.40	0.00	111.00	86.60	9.40	12.00
	25	123.50	114.00	2.00	118.00	105.00	11.00	12.00
	50	131.00	125.00	4.00	129.00	120.00	12.00	12.00
	75	136.00	134.00	8.00	135.50	132.00	12.00	12.00
	90	140.00	139.60	12.00	139.60	139.60	12.00	12.00
	95	142.00	142.00	15.10	142.00	142.00	12.00	12.00
	97	142.38	142.00	20.38	142.00	142.00	12.00	12.00
	99	143.00	142.00	21.00	142.00	142.00	12.00	12.00

TR = total recall; LTR = long term retrieval; STR = short term retrieval; LTS = long term storage; CLTR = consistent long term retrieval; MCR = multiple choice recognition; DR = delayed recall

Table B2

Normative data (percentile ranks) for men aged 30-39, stratified according to education level

Education level	Percentile	TR	LTR	STR	LTS	CLTR	DR	MCR
1	1	47.00	27.00	1.00	43.00	2.00	0.00	7.00
(n = 44)	2	47.00	27.00	1.00	43.00	2.00	0.00	7.00
	5	79.75	42.00	2.00	53.50	9.75	3.50	11.00
	10	88.00	59.50	2.00	73.00	20.00	5.00	11.00
	25	94.50	78.00	5.00	88.75	40.75	8.25	12.00
	50	113.00	104.00	11.00	113.00	79.50	10.00	12.00
	75	122.00	113.75	20.00	121.75	101.25	12.00	12.00
	90	127.50	124.50	25.50	130.00	115.50	12.00	12.00
	95	130.75	127.50	35.25	131.75	127.50	12.00	12.00
	97	132.30	129.95	41.55	133.30	128.65	12.00	12.00
	99	133.00	131.00	44.00	134.00	129.00	12.00	12.00
2	1	45.00	11.00	0.00	24.00	0.00	1.00	11.00
(n = 74)	2	57.50	24.50	0.00	38.00	7.50	3.00	11.00
	5	81.75	52.00	1.00	64.75	19.00	5.75	12.00
	10	89.00	65.00	2.00	74.50	30.00	7.50	12.00
	25	102.00	85.50	5.00	94.00	51.50	9.75	12.00
	50	116.00	108.50	8.00	117.00	85.00	11.00	12.00
	75	128.25	123.00	15.25	128.25	112.75	12.00	12.00
	90	135.00	132.00	27.50	133.00	130.00	12.00	12.00
	95	137.00	133.25	32.50	135.25	131.254	12.00	12.00
	97	137.75	137.00	34.00	137.50	136.50	12.00	12.00
	99	140.00	138.00	39.00	138.00	138.00	12.00	12.00
3	1	81.00	48.00	0.00	53.00	29.00	7.00	10.00
(n = 57)	2	83.88	54.08	0.00	60.04	29.80	7.00	10.00
	5	99.00	91.40	0.90	99.70	38.50	7.00	10.90
	10	104.80	93.80	1.00	105.20	47.80	8.00	11.80
	25	112.00	101.00	2.00	112.00	76.00	10.00	12.00
	50	125.00	118.00	5.00	124.00	108.00	11.00	12.00
	75	131.50	128.00	10.50	131.00	123.50	12.00	12.00
	90	135.20	134.40	16.20	136.00	134.00	12.00	12.00
	95	137.30	136.40	17.10	137.30	136.40	12.00	12.00
	97	140.26	140.00	21.90	140.00	140.00	12.00	12.00
	99	141.00	140.00	33.00	140.00	140.00	12.00	12.00
4	1	89.00	57.00	0.00	66.00	35.00	6.00	11.00
(n = 64)	2	89.30	60.30	0.00	69.90	35.90	6.30	11.00
	5	97.75	84.50	0.00	91.25	45.00	9.00	11.25
	10	105.50	90.00	1.00	100.00	58.00	10.00	12.00
	25	116.00	103.00	4.00	112.00	78.50	11.00	12.00
	50	125.50	119.50	6.50	124.50	105.50	12.00	12.00
	75	133.00	128.75	12.00	132.00	126.75	12.00	12.00
	90	137.00	135.00	16.50	136.50	132.50	12.00	12.00
	95	139.75	139.25	20.00	139.50	139.25	12.00	12.00
	97	141.05	140.10	24.45	140.10	140.10	12.00	12.00
	99	142.00	142.00	33.00	142.00	142.00	12.00	12.00

TR = total recall; LTR = long term retrieval; STR = short term retrieval; LTS = long term storage; CLTR = consistent long term retrieval; MCR = multiple choice recognition; DR = delayed recall

Table B3

Normative data (percentile ranks) for men aged 40-49, stratified according to education level

Education level	Percentile	TR	LTR	STR	LTS	CLTR	DR	MCR
1	1	58.00	15.00	1.00	26.00	0.00	4.00	10.00
(n = 61)	2	61.12	20.04	1.00	30.08	4.80	4.24	10.24
	5	77.10	37.90	2.10	47.70	21.10	5.00	11.00
	10	80.20	50.20	3.20	60.60	23.40	6.20	11.00
	25	95.00	73.00	6.00	85.50	38.50	8.00	12.00
	50	110.00	99.00	12.00	108.00	62.00	10.00	12.00
	75	119.50	112.00	21.50	119.00	100.00	12.00	12.00
	90	129.80	126.00	30.20	129.00	122.60	12.00	12.00
	95	132.00	128.90	39.50	131.90	125.80	12.00	12.00
	97	133.14	131.00	41.28	133.14	126.14	12.00	12.00
	99	134.00	131.00	43.00	134.00	127.00	12.00	12.00
2	1	71.00	13.00	0.00	17.00	4.00	2.00	7.00
(n = 64)	2	71.60	19.30	0.00	23.30	6.10	2.60	7.30
	5	79.50	39.00	1.25	44.25	13.75	5.00	10.25
	10	84.50	52.00	3.50	62.50	24.00	6.00	11.00
	25	94.25	76.25	7.25	87.25	40.75	8.00	12.00
	50	109.00	94.00	14.00	106.50	68.00	10.00	12.00
	75	118.75	108.75	22.00	117.75	97.75	12.00	12.00
	90	132.00	129.00	34.00	129.00	125.50	12.00	12.00
	95	134.00	133.25	45.00	134.00	132.75	12.00	12.00
	97	136.00	134.05	50.40	134.05	134.05	12.00	12.00
	99	136.00	135.00	58.00	135.00	135.00	12.00	12.00
3	1	88.00	52.00	0.00	55.00	19.00	6.00	11.00
(n = 59)	2	88.00	52.60	0.00	56.80	19.20	6.20	11.00
	5	89.00	56.00	0.00	66.00	25.00	7.00	11.00
	10	92.00	67.00	1.00	75.00	33.00	7.00	12.00
	25	101.00	80.00	5.00	94.00	46.00	9.00	12.00
	50	118.00	105.00	10.00	114.00	82.00	11.00	12.00
	75	129.00	125.00	19.00	127.00	117.00	12.00	12.00
	90	134.00	130.00	28.00	132.00	127.00	12.00	12.00
	95	136.00	133.00	33.00	138.00	130.00	12.00	12.00
	97	138.80	138.80	36.00	142.00	134.00	12.00	12.00
	99	142.00	142.00	36.00	142.00	142.00	12.00	12.00
4	1	72.00	32.00	0.00	40.00	8.00	4.00	10.00
(n = 75)	2	78.76	45.52	0.52	58.20	12.68	4.00	10.52
	5	92.20	63.40	1.00	78.80	22.00	6.00	11.00
	10	97.00	76.20	2.00	93.40	33.60	7.60	12.00
	25	109.00	95.00	4.00	111.00	66.00	10.00	12.00
	50	125.00	116.00	7.00	120.00	106.00	12.00	12.00
	75	130.00	126.00	13.00	128.00	122.00	12.00	12.00
	90	135.40	133.00	20.40	132.40	131.00	12.00	12.00
	95	137.20	135.20	27.20	135.00	134.20	12.00	12.00
	97	138.72	136.72	30.16	135.72	135.72	12.00	12.00
	99	140.00	137.00	40.00	137.00	137.00	12.00	12.00

TR = total recall; LTR = long term retrieval; STR = short term retrieval; LTS = long term storage; CLTR = consistent long term retrieval; MCR = multiple choice recognition; DR = delayed recall

Table B4

Normative data (percentile ranks) for men aged 50-59, stratified according to education level

Education level	Percentile	TR	LTR	STR	LTS	CLTR	DR	MCR
1	1	58.00	12.00	3.00	27.00	2.00	3.00	10.00
(n = 56)	2	58.42	13.54	3.14	27.84	2.28	3.14	10.00
	5	69.50	33.20	4.85	41.50	5.70	4.85	10.85
	10	77.00	45.20	5.00	56.70	15.20	6.00	11.00
	25	86.00	57.25	13.25	71.50	29.00	7.25	12.00
	50	96.50	78.50	21.00	88.50	44.00	9.00	12.00
	75	107.25	94.00	27.75	106.25	70.00	11.00	12.00
	90	124.30	116.30	39.30	121.30	108.30	12.00	12.00
	95	126.20	121.20	46.45	128.15	113.55	12.00	12.00
	97	133.29	128.29	49.00	129.58	128.29	12.00	12.00
	99	134.00	129.00	49.00	131.00	129.00	12.00	12.00
2	1	65.00	35.00	1.00	46.00	5.00	3.00	10.00
(n = 79)	2	73.40	39.20	1.60	49.60	6.80	3.60	10.60
	5	81.00	47.00	2.00	57.00	11.00	5.00	11.00
	10	83.00	54.00	4.00	64.00	21.00	6.00	11.00
	25	95.00	74.00	8.00	87.00	36.00	8.00	12.00
	50	103.00	92.00	14.00	106.00	59.00	10.00	12.00
	75	119.00	109.00	22.00	119.00	94.00	12.00	12.00
	90	128.00	123.00	30.00	127.00	115.00	12.00	12.00
	95	130.00	126.00	36.00	129.00	124.00	12.00	12.00
	97	132.60	128.80	36.60	131.80	128.40	12.00	12.00
	99	138.00	136.00	37.00	136.00	136.00	12.00	12.00
3	1	65.00	29.00	0.00	39.00	2.00	4.00	9.00
(n = 92)	2	70.16	34.16	0.00	39.00	6.30	4.00	9.86
	5	81.25	43.90	2.00	55.95	14.60	5.00	10.00
	10	88.00	61.30	4.00	75.00	26.00	5.30	11.00
	25	101.00	77.00	7.00	90.00	47.00	8.00	12.00
	50	109.50	97.00	12.00	107.00	71.00	10.00	12.00
	75	122.00	115.00	21.00	120.00	103.50	11.00	12.00
	90	131.00	126.00	30.00	129.00	121.00	12.00	12.00
	95	135.70	133.70	31.70	136.35	126.35	12.00	12.00
	97	138.21	137.21	38.84	138.00	137.21	12.00	12.00
	99	143.00	143.00	48.00	143.00	143.00	12.00	12.00
4	1	57.00	26.00	0.00	35.00	7.00	2.00	9.00
(n = 93)	2	70.20	36.56	0.00	42.04	8.76	2.88	9.88
	5	75.80	48.40	0.70	63.40	14.70	6.00	11.00
	10	89.20	59.80	2.40	72.80	30.80	8.00	11.00
	25	106.00	89.50	5.00	101.50	56.00	10.00	12.00
	50	119.00	108.00	9.00	118.00	91.00	11.00	12.00
	75	128.50	123.50	17.00	127.50	118.00	12.00	12.00
	90	134.00	131.00	28.20	133.00	127.00	12.00	12.00
	95	136.30	133.00	30.30	134.00	132.30	12.00	12.00
	97	138.18	134.72	35.18	134.72	134.72	12.00	12.00
	99	143.00	143.00	41.00	143.00	143.00	12.00	12.00

TR = total recall; LTR = long term retrieval; STR = short term retrieval; LTS = long term storage; CLTR = consistent long term retrieval; MCR = multiple choice recognition; DR = delayed recall

Table B5

Normative data (percentile ranks) for men aged 60-69, stratified according to education level

Education level	Percentile	TR	LTR	STR	LTS	CLTR	DR	MCR
1	1	42.00	4.00	1.00	10.00	0.00	2.00	8.00
(n = 44)	2	42.00	4.00	1.00	10.00	0.00	2.00	8.00
	5	46.75	11.75	2.25	23.75	0.00	2.00	10.00
	10	53.50	19.50	4.00	30.50	1.50	3.00	10.50
	25	73.25	36.25	12.00	48.00	9.00	5.00	11.25
	50	86.50	63.50	22.50	75.00	35.00	7.00	12.00
	75	100.75	90.50	35.00	99.75	60.00	10.75	12.00
	90	120.50	113.00	39.50	122.50	92.00	12.00	12.00
	95	136.50	134.00	41.75	135.50	132.50	12.00	12.00
	97	137.65	135.30	45.25	136.00	135.30	12.00	12.00
	99	138.00	136.00	47.00	136.00	136.00	12.00	12.00
2	1	49.00	10.00	1.00	14.00	0.00	2.00	9.00
(n = 58)	2	49.90	10.90	1.54	15.62	0.00	2.18	9.18
	5	55.90	19.75	4.95	28.70	1.90	3.00	10.00
	10	61.60	28.50	7.00	42.00	6.00	4.00	11.00
	25	81.00	47.00	11.00	59.25	18.75	6.00	11.00
	50	95.00	80.00	19.00	91.00	40.50	9.00	12.00
	75	109.25	91.50	29.00	108.25	70.00	10.25	12.00
	90	117.00	108.10	36.10	120.10	83.80	12.00	12.00
	95	120.25	112.25	40.15	122.45	102.25	12.00	12.00
	97	126.61	119.53	43.46	131.46	107.92	12.00	12.00
	99	132.00	128.00	45.00	133.00	111.00	12.00	12.00
3	1	21.00	9.00	0.00	18.00	0.00	1.00	9.00
(n = 35)	2	21.00	9.00	0.00	18.00	0.00	1.00	9.00
	5	53.00	10.60	1.60	23.60	1.60	3.40	9.80
	10	70.20	21.80	4.20	28.00	4.80	4.00	11.00
	25	78.00	45.00	11.00	59.00	15.00	6.00	12.00
	50	104.00	84.00	15.00	92.00	54.00	8.00	12.00
	75	119.00	107.00	31.00	112.00	86.00	12.00	12.00
	90	127.00	121.60	43.80	126.40	117.40	12.00	12.00
	95	137.40	137.00	49.60	137.20	135.40	12.00	12.00
	97	138.84	137.00	51.76	137.92	136.84	12.00	12.00
	99	139.00	137.00	52.00	138.00	137.00	12.00	12.00
4	1	58.00	10.00	2.00	17.00	0.00	4.00	11.00
(n = 50)	2	58.16	10.22	2.02	17.24	0.00	4.02	11.00
	5	68.75	30.90	3.00	39.45	3.30	5.00	11.00
	10	81.30	50.60	4.00	67.00	14.30	6.00	11.00
	25	91.75	69.75	7.75	86.00	40.75	8.00	12.00
	50	105.50	94.00	12.50	101.50	62.00	10.50	12.00
	75	115.50	109.25	23.00	117.00	90.00	11.00	12.00
	90	132.20	128.10	30.80	129.60	120.90	12.00	12.00
	95	135.00	132.00	37.85	132.45	128.70	12.00	12.00
	97	135.94	132.94	52.52	134.41	132.47	12.00	12.00
	99	137.00	134.00	61.00	136.00	133.00	12.00	12.00

TR = total recall; LTR = long term retrieval; STR = short term retrieval; LTS = long term storage; CLTR = consistent long term retrieval; MCR = multiple choice recognition; DR = delayed recall

Table B6

Normative data (percentile ranks) for men aged 70 and older, stratified according to education level

Education level	Percentile	TR	LTR	STR	LTS	CLTR	DR	MCR
1	1	34.00	16.00	5.00	22.00	0.00	2.00	4.00
(n = 54)	2	34.30	16.10	5.00	22.50	0.00	2.00	4.30
	5	39.25	17.00	5.00	27.00	0.00	2.00	7.75
	10	54.50	18.00	7.00	29.50	2.00	3.00	9.00
	25	60.75	28.50	13.00	39.50	7.50	4.00	11.00
	50	76.50	51.00	26.50	67.00	19.50	6.00	12.00
	75	93.75	76.00	31.50	89.75	38.75	9.00	12.00
	90	118.00	110.50	37.00	120.00	95.50	10.00	12.00
	95	130.25	123.50	38.25	125.00	120.50	11.25	12.00
	97	132.40	126.75	39.35	126.75	126.75	12.00	12.00
	99	135.00	130.00	40.00	130.00	130.00	12.00	12.00
2	1	41.00	5.00	2.00	6.00	0.00	1.00	5.00
(n = 51)	2	41.28	5.12	2.04	6.20	0.00	1.08	5.16
	5	48.60	11.60	3.60	18.80	0.00	3.00	9.00
	10	56.80	25.20	8.00	34.80	2.20	4.00	11.00
	25	73.00	38.00	11.00	52.00	11.00	4.00	11.00
	50	85.00	61.00	23.00	71.00	33.00	7.00	12.00
	75	101.00	91.00	34.00	101.00	49.00	9.00	12.00
	90	116.80	106.00	41.60	115.80	82.40	10.80	12.00
	95	121.20	115.20	43.80	118.60	108.80	11.40	12.00
	97	124.32	118.76	45.44	128.04	114.76	12.00	12.00
	99	126.00	121.00	46.00	137.00	117.00	12.00	12.00
3	1	43.00	2.00	5.00	4.00	0.00	1.00	5.00
(n = 53)	2	43.54	2.36	5.06	4.54	0.00	1.06	5.18
	5	52.65	9.95	6.65	20.15	0.00	2.00	8.65
	10	63.30	19.60	7.00	32.50	0.00	3.30	11.00
	25	75.25	46.25	12.75	62.50	14.00	5.00	11.25
	50	91.50	70.00	21.00	85.00	36.50	8.00	12.00
	75	104.00	89.75	29.00	101.75	59.75	10.00	12.00
	90	114.40	103.70	42.40	117.10	83.00	11.00	12.00
	95	122.05	115.05	47.75	121.70	99.35	12.00	12.00
	97	126.05	119.05	51.82	123.41	106.56	12.00	12.00
	99	129.00	122.00	53.00	124.00	116.00	12.00	12.00
4	1	30.00	5.00	1.00	8.00	0.00	0.00	6.00
(n = 59)	2	30.20	5.20	1.00	8.20	0.00	0.00	6.80
	5	50.00	10.00	2.00	18.00	0.00	1.00	10.00
	10	58.00	21.00	2.00	36.00	4.00	3.00	11.00
	25	81.00	52.00	7.00	63.00	18.00	6.00	12.00
	50	92.00	71.00	17.00	92.00	49.00	9.00	12.00
	75	122.00	112.00	28.00	120.00	101.00	11.00	12.00
	90	129.00	127.00	36.00	129.00	120.00	12.00	12.00
	95	132.00	130.00	46.00	136.00	130.00	12.00	12.00
	97	138.40	136.60	47.20	137.40	136.60	12.00	12.00
	99	140.00	139.00	52.00	139.00	139.00	12.00	12.00

TR = total recall; LTR = long term retrieval; STR = short term retrieval; LTS = long term storage; CLTR = consistent long term retrieval; MCR = multiple choice recognition; DR = delayed recall

Table B7

Normative data (percentile ranks) for women with aged 18-29, stratified according to education level

Education level	Percentile	TR	LTR	STR	LTS	CLTR	DR	MCR
1	1	76.00	17.00	0.00	67.00	14.00	6.00	10.00
(n = 62)	2	76.52	26.62	0.26	67.52	14.52	6.00	10.25
	5	84.45	56.05	1.00	70.00	24.05	7.15	11.00
	10	95.60	70.30	1.30	88.90	43.60	8.00	12.00
	25	105.75	93.75	4.00	103.75	55.75	10.00	12.00
	50	121.00	111.50	7.00	119.50	94.00	11.00	12.00
	75	130.25	125.25	14.00	127.25	122.00	12.00	12.00
	90	135.00	134.00	20.40	135.70	130.10	12.00	12.00
	95	138.00	135.85	29.95	137.85	135.85	12.00	12.00
	97	140.11	140.00	41.49	140.00	140.00	12.00	12.00
	99	141.00	140.00	94.00	140.00	140.00	12.00	12.00
2	1	84.10	56.35	0.00	64.80	30.75	6.70	11.35
(n = 134)	2	88.00	66.80	0.00	75.60	37.50	8.00	12.00
	5	101.75	85.50	0.00	95.00	49.25	9.00	12.00
	10	108.00	93.50	1.50	104.00	63.00	10.00	12.00
	25	117.00	106.00	3.00	114.25	84.00	11.00	12.00
	50	127.00	121.00	6.00	125.00	111.00	12.00	12.00
	75	134.00	131.00	11.00	133.00	127.25	12.00	12.00
	90	138.00	136.00	16.00	136.50	134.00	12.00	12.00
	95	140.00	139.25	19.25	140.00	138.00	12.00	12.00
	97	141.00	140.00	24.75	141.00	139.95	12.00	12.00
	99	142.00	142.00	29.60	143.65	141.65	12.00	12.00
3	1	99.94	70.88	0.00	40.93	49.47	6.49	8.45
(n = 148)	2	103.00	82.88	0.00	80.78	63.74	7.98	11.00
	5	114.45	97.90	0.00	101.80	72.00	9.00	12.00
	10	117.90	106.90	1.00	110.00	80.80	10.90	12.00
	25	124.00	116.00	2.00	121.00	101.00	11.00	12.00
	50	131.00	126.00	5.00	128.00	117.50	12.00	12.00
	75	135.00	132.00	8.00	134.00	129.00	12.00	12.00
	90	139.00	138.00	12.00	138.00	137.10	12.00	12.00
	95	140.00	139.55	16.10	140.00	139.00	12.00	12.00
	97	141.00	140.00	20.00	140.00	140.00	12.00	12.00
	99	142.53	142.53	29.06	142.53	142.53	12.00	12.00
4	1	94.00	69.00	0.00	81.00	42.00	6.00	11.00
(n = 75)	2	97.64	76.28	0.00	88.80	45.64	6.52	11.00
	5	112.60	99.20	0.00	108.20	74.20	9.00	11.80
	10	121.60	112.60	0.60	116.20	88.00	10.60	12.00
	25	128.00	122.00	2.00	125.00	114.00	12.00	12.00
	50	134.00	130.00	4.00	132.00	128.00	12.00	12.00
	75	137.00	137.00	7.00	136.00	135.00	12.00	12.00
	90	140.40	140.00	9.40	140.40	138.00	12.00	12.00
	95	142.00	141.00	14.00	141.00	141.00	12.00	12.00
	97	142.00	141.72	20.16	141.72	141.72	12.00	12.00
	99	143.00	143.00	25.00	143.00	143.00	12.00	12.00

TR = total recall; LTR = long term retrieval; STR = short term retrieval; LTS = long term storage; CLTR = consistent long term retrieval; MCR = multiple choice recognition; DR = delayed recall

Table B8

Normative data (percentile ranks) for women aged 30-39, stratified according to education level

Education level	Percentile	TR	LTR	STR	LTS	CLTR	DR	MCR
1	1	73.00	42.00	0.00	65.00	4.00	6.00	10.00
(n = 28)	2	73.00	42.00	0.00	65.00	4.00	6.00	10.00
	5	77.05	51.00	0.90	67.25	9.85	6.00	10.45
	10	92.80	67.40	2.00	79.00	35.90	7.80	11.00
	25	111.00	97.00	4.25	107.25	64.75	10.00	12.00
	50	115.50	108.00	7.50	118.50	91.00	11.00	12.00
	75	125.75	122.75	11.75	124.75	108.50	12.00	12.00
	90	130.20	127.30	27.40	133.20	123.00	12.00	12.00
	95	132.55	130.00	31.55	135.00	123.55	12.00	12.00
	97	133.00	130.00	32.00	135.00	124.00	12.00	12.00
	99	133.00	130.00	32.00	135.00	124.00	12.00	12.00
2	1	91.00	60.00	1.00	75.00	18.00	5.00	10.00
(n = 60)	2	91.88	60.88	1.00	75.44	21.08	5.44	10.22
	5	95.20	74.25	1.00	77.20	34.05	8.00	11.05
	10	102.10	82.30	2.00	94.10	49.40	9.00	12.00
	25	108.25	95.50	3.00	105.25	73.75	10.00	12.00
	50	121.00	113.00	7.00	119.50	95.00	12.00	12.00
	75	130.00	125.75	13.75	128.75	118.00	12.00	12.00
	90	133.00	129.90	19.70	133.00	127.90	12.00	12.00
	95	133.00	131.95	27.65	135.00	129.00	12.00	12.00
	97	137.02	135.19	31.00	137.68	133.53	12.00	12.00
	99	142.00	141.00	31.00	141.00	141.00	12.00	12.00
3	1	80.00	48.00	0.00	64.00	23.00	7.00	11.00
(n = 58)	2	82.34	51.42	0.00	66.16	26.42	7.36	11.18
	5	103.45	88.85	0.00	101.65	53.40	9.00	12.00
	10	110.90	96.80	1.00	105.90	59.70	9.90	12.00
	25	115.00	106.00	2.00	115.00	79.00	11.00	12.00
	50	125.50	121.00	5.00	124.00	105.50	12.00	12.00
	75	136.00	134.50	10.00	135.00	132.25	12.00	12.00
	90	139.00	138.00	14.00	139.00	137.00	12.00	12.00
	95	140.00	139.05	16.50	140.00	138.10	12.00	12.00
	97	140.46	140.00	27.38	140.00	140.00	12.00	12.00
	99	142.00	140.00	32.00	140.00	140.00	12.00	12.00
4	1	96.00	64.00	0.00	75.00	24.00	6.00	11.00
(n = 74)	2	96.50	70.50	0.00	82.50	25.00	7.00	11.00
	5	100.25	87.75	0.00	100.50	44.25	8.00	11.75
	10	113.00	102.50	1.00	110.50	67.00	10.00	12.00
	25	123.00	116.00	2.75	121.50	99.00	11.00	12.00
	50	130.00	125.00	5.00	127.00	118.50	12.00	12.00
	75	134.25	131.25	7.00	133.25	128.25	12.00	12.00
	90	137.50	135.50	11.50	136.50	135.50	12.00	12.00
	95	141.50	141.50	15.25	141.50	141.00	12.00	12.00
	97	143.00	143.00	22.00	143.75	142.50	12.00	12.00
	99	144.00	144.00	33.00	144.00	144.00	12.00	12.00

TR = total recall; LTR = long term retrieval; STR = short term retrieval; LTS = long term storage; CLTR = consistent long term retrieval; MCR = multiple choice recognition; DR = delayed recall

Table B9

Normative data (percentile ranks) for women aged 40-49, stratified according to education level

Education level	Percentile	TR	LTR	STR	LTS	CLTR	DR	MCR
1	1	71.00	41.00	0.00	59.00	14.00	3.00	10.00
(n = 66)	2	75.08	46.10	0.34	61.72	16.04	4.02	10.34
	5	92.35	68.80	1.00	82.05	23.05	7.00	11.00
	10	97.00	76.40	2.00	85.00	31.10	8.00	12.00
	25	104.00	92.00	5.00	99.75	56.25	9.00	12.00
	50	116.00	106.50	9.00	116.00	75.50	11.00	12.00
	75	127.00	121.00	16.00	125.25	114.00	12.00	12.00
	90	131.30	129.30	23.00	132.00	121.60	12.00	12.00
	95	133.65	132.60	27.30	136.65	127.30	12.00	12.00
	97	137.96	135.98	29.98	137.99	128.99	12.00	12.00
	99	138.00	137.00	36.00	140.00	132.00	12.00	12.00
2	1	63.00	46.00	0.00	63.00	19.00	3.00	8.00
(n = 69)	2	70.60	48.80	0.00	64.60	20.60	4.20	9.20
	5	91.00	65.50	0.50	72.50	36.00	7.00	11.50
	10	98.00	75.00	2.00	86.00	46.00	7.00	12.00
	25	110.00	97.50	4.00	103.50	58.50	10.00	12.00
	50	122.00	115.00	8.00	121.00	95.00	11.00	12.00
	75	128.50	124.50	15.00	128.00	113.50	12.00	12.00
	90	135.00	131.00	23.00	133.00	129.00	12.00	12.00
	95	135.50	135.50	28.00	136.00	133.00	12.00	12.00
	97	136.90	136.00	31.70	137.80	135.70	12.00	12.00
	99	138.00	138.00	34.00	138.00	138.00	12.00	12.00
3	1	87.00	56.00	0.00	60.00	34.00	7.00	6.00
(n = 68)	2	88.14	60.56	0.38	68.74	35.52	7.00	7.90
	5	94.35	77.35	1.00	94.90	40.80	8.00	12.00
	10	106.00	92.50	2.00	99.80	56.00	9.00	12.00
	25	116.00	106.25	4.00	112.25	74.75	11.00	12.00
	50	124.50	117.00	7.00	123.00	102.50	12.00	12.00
	75	132.00	127.75	11.75	129.75	122.00	12.00	12.00
	90	135.00	133.00	16.20	134.00	130.10	12.00	12.00
	95	136.00	134.00	21.10	136.10	133.55	12.00	12.00
	97	136.93	134.93	27.58	139.79	134.00	12.00	12.00
	99	140.00	140.00	31.00	140.00	140.00	12.00	12.00
4	1	98.00	57.00	0.00	64.00	14.00	7.00	11.00
(n = 59)	2	99.20	62.40	0.00	70.60	20.00	7.20	11.00
	5	104.00	88.00	0.00	97.00	51.00	10.00	11.00
	10	110.00	95.00	1.00	104.00	61.00	10.00	12.00
	25	119.00	109.00	3.00	113.00	91.00	11.00	12.00
	50	127.00	119.00	7.00	123.00	113.00	12.00	12.00
	75	134.00	131.00	12.00	132.00	128.00	12.00	12.00
	90	139.00	138.00	16.00	139.00	138.00	12.00	12.00
	95	142.00	142.00	16.00	142.00	142.00	12.00	12.00
	97	142.40	142.40	24.20	143.20	142.40	12.00	12.00
	99	144.00	144.00	41.00	144.00	144.00	12.00	12.00

TR = total recall; LTR = long term retrieval; STR = short term retrieval; LTS = long term storage; CLTR = consistent long term retrieval; MCR = multiple choice recognition; DR = delayed recall

Table B10

Normative data (percentile ranks) for women aged 50-59, stratified according to education level

Education level	Percentile	TR	LTR	STR	LTS	CLTR	DR	MCR
1	1	52.00	13.00	2.00	18.00	7.00	1.00	10.00
(n = 63)	2	56.48	20.28	2.00	26.68	8.68	2.40	10.28
	5	75.40	40.20	3.00	50.20	16.00	6.20	11.00
	10	79.20	48.20	3.40	57.00	20.80	7.00	11.00
	25	89.00	66.00	9.00	77.00	36.00	8.00	12.00
	50	106.00	89.00	13.00	102.00	64.00	10.00	12.00
	75	119.00	111.00	22.00	117.00	98.00	12.00	12.00
	90	128.40	124.60	33.60	127.60	114.80	12.00	12.00
	95	132.60	129.40	36.60	133.00	121.00	12.00	12.00
	97	134.00	131.08	38.08	133.24	125.40	12.00	12.00
	99	134.00	132.00	39.00	136.00	130.00	12.00	12.00
2	1	54.00	7.00	0.00	10.00	0.00	3.00	11.00
(n = 66)	2	60.46	13.12	0.00	15.78	6.46	3.68	11.00
	5	86.10	57.05	0.35	67.10	29.00	7.00	11.00
	10	95.80	73.40	2.00	87.70	39.40	8.70	12.00
	25	106.75	89.00	4.00	97.75	60.00	9.00	12.00
	50	119.00	112.00	7.00	121.50	88.50	12.00	12.00
	75	128.00	123.25	18.00	127.00	115.25	12.00	12.00
	90	134.00	130.60	25.60	134.00	125.30	12.00	12.00
	95	136.65	136.30	30.30	137.00	136.30	12.00	12.00
	97	138.98	138.98	46.84	138.98	138.98	12.00	12.00
	99	142.00	141.00	48.00	141.00	141.00	12.00	12.00
3	1	94.00	74.00	1.00	88.00	32.00	5.00	9.00
(n = 80)	2	94.00	75.86	1.62	89.24	33.24	5.62	10.24
	5	101.00	82.05	2.00	92.00	50.05	7.05	12.00
	10	103.00	84.20	3.00	95.10	52.00	9.00	12.00
	25	113.00	102.50	5.00	111.25	73.25	10.00	12.00
	50	123.50	115.50	7.50	121.00	100.00	12.00	12.00
	75	129.00	124.75	11.75	127.00	115.75	12.00	12.00
	90	134.80	130.00	18.00	131.90	128.00	12.00	12.00
	95	136.00	133.95	20.95	135.00	130.90	12.00	12.00
	97	137.00	134.57	21.00	135.57	133.57	12.00	12.00
	99	138.00	137.00	22.00	139.00	135.00	12.00	12.00
4	1	97.00	68.00	0.00	73.00	35.00	6.00	10.00
(n = 93)	2	97.88	68.88	0.00	78.28	38.52	6.88	10.88
	5	102.00	83.80	0.70	94.80	50.70	8.00	11.70
	10	109.40	93.80	1.40	101.40	56.20	9.00	12.00
	25	119.50	108.00	3.00	111.00	91.00	11.00	12.00
	50	128.00	122.00	6.00	124.00	114.00	12.00	12.00
	75	134.00	130.00	11.50	132.50	126.00	12.00	12.00
	90	137.00	135.00	17.00	136.00	133.60	12.00	12.00
	95	139.00	137.30	20.00	138.30	135.30	12.00	12.00
	97	139.36	139.36	22.44	139.36	139.36	12.00	12.00
	99	142.00	141.00	34.00	141.00	141.00	12.00	12.00

TR = total recall; LTR = long term retrieval; STR = short term retrieval; LTS = long term storage; CLTR = consistent long term retrieval; MCR = multiple choice recognition; DR = delayed recall

Table B11

Normative data (percentile ranks) for women aged 60-69, stratified according to education level

Education level	Percentile	TR	LTR	STR	LTS	CLTR	DR	MCR
1	1	57.00	13.00	1.00	19.00	2.00	3.00	10.00
(n = 52)	2	57.06	13.84	1.18	19.72	2.30	3.06	10.00
	5	58.65	28.95	4.00	36.85	9.60	4.00	10.65
	10	71.20	43.60	5.00	57.10	12.30	5.00	11.00
	25	84.00	57.25	10.25	69.00	31.75	7.00	12.00
	50	101.00	80.50	19.00	93.00	47.50	9.50	12.00
	75	113.00	102.75	25.75	111.00	80.50	11.00	12.00
	90	127.20	120.70	31.70	124.20	104.10	12.00	12.00
	95	130.35	126.35	38.15	130.35	115.40	12.00	12.00
	97	131.00	127.41	44.82	132.23	120.05	12.00	12.00
	99	131.00	128.00	46.00	134.00	123.00	12.00	12.00
2	1	52.00	2.00	4.00	2.00	0.00	0.00	9.00
(n = 55)	2	52.60	3.32	4.00	3.44	0.24	0.36	9.12
	5	61.00	21.80	4.80	39.60	3.60	3.00	10.00
	10	68.60	33.60	6.60	46.60	11.40	4.00	11.00
	25	76.00	49.00	10.00	66.00	18.00	7.00	12.00
	50	100.00	81.00	18.00	93.00	52.00	9.00	12.00
	75	118.00	108.00	28.00	115.00	86.00	11.00	12.00
	90	125.00	116.80	35.40	123.00	102.60	12.00	12.00
	95	126.20	121.20	46.20	125.40	111.20	12.00	12.00
	97	128.28	123.60	56.92	127.96	112.00	12.00	12.00
	99	131.00	127.00	61.00	130.00	112.00	12.00	12.00
3	1	69.00	43.00	0.00	52.00	6.00	5.00	7.00
(n = 46)	2	69.00	43.00	0.00	52.00	6.00	5.00	7.00
	5	72.35	46.40	2.35	61.70	14.05	5.00	11.00
	10	82.20	54.00	5.10	69.60	24.80	6.70	11.00
	25	96.00	75.00	9.75	84.25	40.75	9.00	12.00
	50	106.00	91.50	13.00	104.50	53.00	10.00	12.00
	75	119.25	109.50	21.25	115.50	88.75	11.00	12.00
	90	126.50	120.30	28.60	126.20	110.10	12.00	12.00
	95	132.95	130.00	31.30	131.00	127.30	12.00	12.00
	97	136.95	130.59	34.95	131.00	129.77	12.00	12.00
	99	139.00	131.00	37.00	131.00	131.00	12.00	12.00
4	1	68.00	35.00	1.00	47.00	6.00	3.00	10.00
(n = 49)	2	68.00	35.00	1.00	47.00	6.00	3.00	10.00
	5	76.00	52.00	2.00	71.50	11.50	4.50	11.00
	10	85.00	63.00	3.00	84.00	25.00	7.00	12.00
	25	102.00	85.50	5.00	96.50	48.50	9.00	12.00
	50	114.00	105.00	11.00	114.00	75.00	11.00	12.00
	75	127.50	124.00	16.50	127.50	110.00	12.00	12.00
	90	135.00	130.00	23.00	133.00	126.00	12.00	12.00
	95	135.50	133.00	28.00	134.50	130.00	12.00	12.00
	97	136.50	134.00	31.50	136.00	131.50	12.00	12.00
	99	137.00	135.00	33.00	137.00	133.00	12.00	12.00

TR = total recall; LTR = long term retrieval; STR = short term retrieval; LTS = long term storage; CLTR = consistent long term retrieval; MCR = multiple choice recognition; DR = delayed recall

Table B12

Normative data (percentile ranks) for women aged 70 and older, stratified according to education level

Education level	Percentile	TR	LTR	STR	LTS	CLTR	DR	MCR
1	1	37.00	9.00	1.00	15.00	0.00	2.00	4.00
(n = 61)	2	38.44	10.20	1.48	16.44	0.00	2.00	4.48
	5	56.00	15.60	4.10	24.00	2.20	3.10	9.10
	10	60.20	23.80	6.20	34.80	4.20	4.00	10.20
	25	72.50	46.00	14.00	55.00	18.50	6.00	11.00
	50	89.00	66.00	23.00	79.00	34.00	8.00	12.00
	75	102.50	87.50	29.00	100.50	60.50	10.00	12.00
	90	122.80	116.80	34.00	121.80	106.20	12.00	12.00
	95	130.80	124.60	39.90	126.90	120.70	12.00	12.00
	97	132.70	127.98	43.98	128.98	127.70	12.00	12.00
	99	137.00	134.00	50.00	135.00	132.00	12.00	12.00
2	1	43.00	9.00	3.00	9.00	0.00	1.00	7.00
(n = 58)	2	43.18	9.54	3.18	10.80	0.00	1.18	7.36
	5	49.70	14.85	5.90	23.75	0.00	2.00	9.95
	10	54.70	22.90	8.70	31.80	3.90	3.90	10.90
	25	70.50	38.75	12.75	50.00	15.00	6.00	11.00
	50	90.50	61.50	21.50	77.00	40.50	8.00	12.00
	75	103.00	90.00	30.25	102.25	64.50	11.00	12.00
	90	118.30	104.50	39.10	114.10	96.60	12.00	12.00
	95	123.05	118.05	46.00	121.05	113.00	12.00	12.00
	97	124.69	120.15	46.23	122.46	115.53	12.00	12.00
	99	127.00	124.00	47.00	124.00	124.00	12.00	12.00
3	1	48.00	2.00	0.00	2.00	2.00	2.00	5.00
(n = 54)	2	49.60	4.00	0.10	4.90	2.00	2.10	5.50
	5	67.00	29.50	1.75	43.75	2.75	3.00	10.75
	10	73.00	40.00	3.00	52.00	13.00	4.50	11.00
	25	87.50	65.75	8.75	81.75	27.75	7.00	12.00
	50	100.00	82.50	15.50	99.00	48.00	9.50	12.00
	75	117.50	105.00	21.25	112.75	87.25	11.00	12.00
	90	128.00	125.50	34.00	128.00	121.00	12.00	12.00
	95	133.25	128.75	42.25	131.75	128.75	12.00	12.00
	97	134.70	132.05	44.05	134.00	131.70	12.00	12.00
	99	136.00	134.00	46.00	134.00	133.00	12.00	12.00
4	1	40.00	5.00	0.00	12.00	0.00	2.00	6.00
(n = 52)	2	40.24	5.78	0.00	13.14	0.00	2.06	6.12
	5	51.80	19.95	1.30	32.30	1.30	3.00	9.30
	10	63.30	30.20	3.00	39.60	6.30	5.00	10.30
	25	84.25	61.75	6.25	69.00	34.00	8.00	12.00
	50	108.50	94.00	13.50	106.50	63.00	10.00	12.00
	75	120.00	113.00	22.25	119.75	98.75	11.00	12.00
	90	128.40	124.50	37.80	127.70	121.40	12.00	12.00
	95	134.35	131.70	41.40	136.05	126.70	12.00	12.00
	97	137.05	135.87	45.23	138.82	132.92	12.00	12.00
	99	140.00	140.00	47.00	140.00	140.00	12.00	12.00

TR = total recall; LTR = long term retrieval; STR = short term retrieval; LTS = long term storage; CLTR = consistent long term retrieval; MCR = multiple choice recognition; DR = delayed recall

Appendix C. Supplementary tables.

Table C1

Means and standard deviations of the dependent measures stratified by age and education level for men (n = 1627)

Dependent variable	Education level	Age category 18-29	30-39	40-49	50-59	60-69	70+
TR	1	117 (15.0)	109 (17.4)	107 (17.7)	98 (17.1)	86 (24.3)	79.6 (24.1)
Mean(*SD*)	2	121 (13.4)	113 (18.4)	108 (16.9)	106 (15.9)	93 (19.9)	86.4 (20.8)
	3	125 (11.7)	122 (12.3)	115 (15.1)	110 (16.3)	98 (25.1)	89.3 (19.7)
	4	129 (9.4)	123 (12.3)	119 (14.9)	115 (17.8)	105 (18.3)	95.9 (26.7)
LTR	1	106 (22.4)	95 (25.3)	92 (27.4)	77 (26.7)	64 (34.8)	56 (32.2)
Mean(*SD*)	2	112 (19.6)	101 (26.7)	92 (27.8)	90 (24.8)	72 (29.4)	63 (30.6)
	3	118 (17.2)	115 (17.4)	102 (24.2)	95 (25.4)	77 (36.0)	67 (30.0)
	4	123 (13.8)	115 (17.8)	110 (21.6)	103 (26.2)	89 (28.3)	77 (37.1)
STR	1	11 (8.3)	13 (9.9)	15 (10.6)	21 (11.8)	22 (12.8)	24 (10.7)
Mean(*SD*)	2	8.8 (7.0)	11 (9.5)	16 (12.6)	16 (10.1)	20 (11.1)	23 (12.5)
	3	7 (6.4)	7 (6.3)	13 (9.8)	15 (10.2)	21 (14.0)	23 (12.2)
	4	5 (4.9)	8 (6.4)	10 (7.8)	12 (9.3)	16 (11.7)	18 (13.0)
LTS	1	111 (20.8)	105 (22.6)	101 (25.0)	87 (24.8)	76 (33.6)	68 (31.5)
Mean(*SD*)	2	118 (16.4)	110 (23.5)	100 (26.3)	101 (22.7)	85 (29.1)	76 (31.7)
	3	122 (17.9)	120 (14.3)	109 (21.4)	104 (22.7)	85 (33.9)	79 (29.4)
	4	126 (12.0)	120 (15.0)	116 (17.4)	110 (23.2)	98 (26.2)	88 (35.1)
CLTR	1	90 (30.5)	72 (36.0)	69 (35.2)	52 (32.8)	42 (36.0)	31 (34.6)
Mean(*SD*)	2	94 (31.0)	81 (36.9)	70 (36.0)	64 (33.4)	45 (30.3)	37 (29.7)
	3	106 (26.5)	99 (31.0)	80 (36.6)	73 (34.9)	56 (40.7)	40 (29.6)
	4	116 (23.6)	101 (28.5)	93 (35.1)	85 (36.3)	64 (35.3)	55 (42.7)
MCR	1	12 (0.4)	12 (0.8)	12 (0.4)	12 (0.5)	12 (0.8)	11 (1.5)
Mean(*SD*)	2	12 (0.2)	12 (0.2)	12 (0.9)	12 (0.4)	12 (0.6)	11 (1.2)
	3	12 (0.1)	12 (0.4)	12 (0.3)	12 (0.6)	12 (0.7)	12 (1.2)
	4	12 (0.3)	12 (0.2)	12 (0.3)	12 (0.5)	12 (0.4)	12 (0.9)
DR	1	11 (1.9)	10 (2.7)	10 (2.2)	9 (2.2)	7 (3.1)	7 (2.7)
Mean(*SD*)	2	11 (1.5)	11 (2.2)	10 (2.5)	10 (2.4)	8 (2.7)	7 (2.7)
	3	11 (1.3)	11 (1.6)	10 (1.8)	10 (2.3)	8 (3.1)	8 (3.0)
	4	11 (1.3)	11 (1.2)	11 (1.9)	10 (2.0)	10 (2.2)	8 (3.4)

TR = total recall; LTR = long term retrieval; STR = short term retrieval; LTS = long term storage; CLTR = consistent long term retrieval; MCR = multiple choice recognition; DR = delayed recall

Table C2

Means and standard deviations of the dependent measures stratified by age and education level for women (n = 1630)

		Age category					
Dependent variable	Education level	18-29	30-39	40-49	50-59	60-69	70+
TR	1	117 (15.9)	115 (15)	115 (13.7)	104 (18.6)	99 (19.4)	89 (22.6)
Mean(*SD*)	2	125 (12.0)	120 (12.1)	119 (14.4)	116 (16.0)	98 (21.8)	87 (21.9)
	3	129 (8.3)	125 (12.7)	123 (11.6)	121 (11.0)	106 (16.6)	100 (20.8)
	4	132 (8.8)	127 (10.9)	126 (10.9)	125 (10.8)	113 (17.7)	101 (25.1)
LTR	1	106 (24.7)	105 (21.9)	104 (20.6)	88 (27.9)	80 (28.1)	68 (31.8)
Mean(*SD*)	2	117 (17.3)	110 (18.8)	108 (21.3)	105 (26.0)	78 (32.3)	65 (31.4)
	3	123 (13.0)	118 (18.4)	114 (17.2)	112 (15.9)	90 (23.6)	84 (29.7)
	4	127 (13.1)	122 (15.4)	118 (17.0)	118 (16.9)	101 (24.6)	86 (35.2)
STR	1	11 (13.0)	10 (8.9)	11 (7.8)	16 (10.4)	19 (10.5)	21 (10.9)
Mean(*SD*)	2	7 (6.1)	9 (7.3)	10 (8.0)	11 (10.6)	20 (12.7)	22 (11.4)
	3	6 (5.1)	7 (6.2)	9 (6.2)	9 (5.5)	16 (8.3)	17 (11.0)
	4	5 (4.6)	6 (5.3)	8 (6.8)	8 (6.6)	12 (7.9)	15 (11.9)
LTS	1	115 (18.4)	113 (18.1)	112 (17.6)	97 (26.0)	90 (26.9)	79 (30.4)
Mean(*SD*)	2	122 (14.7)	116 (16.1)	114 (18.4)	112 (24.3)	88 (30.7)	76 (30.6)
	3	125 (14.7)	123 (15.1)	120 (14.1)	118 (12.8)	101 (20.7)	95 (27.4)
	4	129 (11.0)	125 (11.9)	122 (14.6)	121 (14.2)	110 (20.1)	95 (33.1)
CLTR	1	88 (34.7)	84 (32.1)	80 (33.5)	65 (34.5)	56 (32.6)	44 (34.7)
Mean(*SD*)	2	104 (27.9)	93 (29.2)	89 (31.5)	86 (33.1)	55 (34.5)	42 (32.5)
	3	114 (20.8)	103 (30.0)	98 (28.3)	94 (26.9)	62 (32.7)	56 (37.4)
	4	121 (20.5)	110 (28.0)	105 (29.3)	106 (27.0)	78 (36.6)	65 (40.1)
MCR	1	12 (0.4)	12 (0.5)	12 (0.3)	12 (0.4)	12 (0.5)	11 (1.4)
Mean(*SD*)	2	12 (0.1)	12 (0.3)	12 (0.5)	12 (0.3)	12 (0.6)	12 (0.9)
	3	12 (0.5)	12 (0.1)	12 (0.7)	12 (0.4)	12 (0.8)	12 (1.0)
	4	12 (0.2)	12 (0.2)	12 (0.3)	12 (0.3)	12 (0.3)	12 (1.1)
DR	1	12 (1.6)	11 (1.8)	10 (1.8)	10 (2.1)	9 (2.6)	8 (2.7)
Mean(*SD*)	2	11 (1.1)	11 (1.5)	11 (1.9)	11 (1.9)	9 (2.9)	8 (3.0)
	3	12 (1.0)	11 (1.1)	11 (1.3)	11 (1.6)	10 (1.9)	9 (2.8)
	4	12 (1.1)	11 (1.2)	11 (1.1)	11 (1.3)	10 (2.3)	9 (2.7)

TR = total recall; LTR = long term retrieval; STR = short term retrieval; LTS = long term storage; CLTR = consistent long term retrieval; MCR = multiple choice recognition; DR = delayed recall

Table C3

Robust regression for total recall

	Unstandardized coefficients		*t*	*p*
	β	Std. Error		
(Constant)	118.68	2.49	47.74	$p < 0.01$**
Age	0.16	0.10	1.53	0.13
Age^2	-0.01	0.001	-9.06	$p < 0.01$**
Sex	0.45	1.39	0.33	0.75
Education level 2	3.65	2.18	1.67	0.09
Education level 3	6.23	2.03	3.06	$p < 0.01$**
Education level 4	6.02	2.41	2.50	0.01*
Age * Sex	0.14	0.04	3.90	$p < 0.01$**
Age * Education Level 2	0.03	0.05	0.54	0.59
Age * Education Level 3	0.09	0.05	1.88	0.06
Age * Education Level 4	0.20	0.06	3.41	$p < 0.01$**

$p < 0.01$ ** , $p < 0.05$ *

Table C4

Robust regression for long term retrieval

	Unstandardized coefficients		*t*	*p*
	B	Std. Error		
(Constant)	110.06	3.62	30.40	$p < 0.01$**
Age	0.19	0.15	1.25	0.21
Age^2	-0.01	0.001	-8.77	$p < 0.01$**
Sex	0.69	2.03	0.34	0.73
Education Level 2	4.81	3.17	1.52	0.13
Education Level 3	7.97	2.97	2.69	$p < 0.01$**
Education level 4	7.99	3.48	2.29	0.02*
Age * Sex	0.19	0.05	3.69	$p < 0.01$**
Age * Education Level 2	0.05	0.08	0.61	0.55
Age * Education Level 3	0.15	0.07	2.04	0.04*
Age * Education Level 4	0.29	0.08	3.43	$p < 0.01$**

$p < 0.01$ **, $p < 0.05$ *

Table C5

Robust regression for short term retrieval

	Unstandardized coefficients		*t*	*p*
	β	Std. Error		
(Constant)	7.75	1.22	6.36	$p < 0.01$**
Age	-0.001	0.05	-0.01	0.99
Age^2	0.003	0.001	5.83	$p < 0.01$**
Sex	-0.30	0.70	-0.44	0.66
Education Level 2	-0.88	1.12	-0.78	0.43
Education Level 3	-1.62	1.03	-1.58	0.11
Education level 4	-1.11	1.16	-0.96	0.34
Age * Sex	-0.05	0.02	-2.62	$p < 0.01$**
Age * Education Level 2	-0.02	0.03	-0.68	0.50
Age * Education Level 3	-0.05	0.03	-1.84	0.07
Age * Education Level 4	-0.10	0.03	-3.58	$p < 0.01$**

$p < 0.01$ **

Table C6

Robust regression for long term storage

	Unstandardized coefficients		*t*	*p*
	β	Std. Error		
(Constant)	117.73	3.23	36.42	$p < 0.01$**
Age	0.14	0.14	1.02	0.31
Age^2	-0.01	0.001	-7.73	$p < 0.01$**
Sex	-0.10	1.80	-0.05	0.96
Education Level 2	1.99	2.95	0.68	0.50
Education Level 3	3.56	2.70	1.32	0.19
Education level 4	1.41	3.13	0.45	0.65
Age * Sex	0.16	0.05	3.38	$p < 0.01$**
Age * Education Level 2	0.10	0.08	1.31	0.19
Age * Education Level 3	0.20	0.07	2.87	$p < 0.01$**
Age * Education Level 4	0.35	0.08	4.46	$p < 0.01$**

$p < 0.01$ **

Table C7

Robust regression for consistent long term retrieval

	Unstandardized coefficients		*t*	*p*
	β	Std. Error		
(Constant)	105.89	4.90	21.61	$p < 0.01$**
Age	-0.64	0.24	-2.72	$p < 0.01$**
Age^2	-0.01	0.002	-2.50	0.01*
Sex	-10.97	6.45	-1.70	0.09
Education level 2	7.65	1.77	4.31	$p < 0.01$**
Education level 3	18.17	1.75	10.41	$p < 0.01$**
Education level 4	29.35	1.81	16.24	$p < 0.01$**
Age * Sex	1.00	0.32	3.12	$p < 0.01$**
Age^2 * Sex	-0.01	0.003	-2.63	$p < 0.01$**

$p < 0.01$ **, $p < 0.05$ *

Table C8

Robust regression for delayed recall

	Unstandardized coefficients		*t*	*p*
	β	Std. Error		
(Constant)	10.18	0.58	17.70	$p < 0.01$**
Age	0.07	0.03	2.06	0.04*
Age^2	-0.001	0.0004	-3.70	$p < 0.01$**
Sex	-0.27	0.25	-1.06	0.29
Education level 2	0.16	0.72	0.23	0.82
Education level 3	1.72	0.66	2.59	$p < 0.01$**
Education level 4	1.68	0.68	2.47	0.01*
Age * Sex	0.02	0.01	4.28	$p < 0.01$**
Age * Education Level 2	0.03	0.04	0.58	0.56
Age * Education Level 3	-0.07	0.04	-1.89	0.06
Age * Education Level 4	-0.06	0.04	-1.50	0.13
Age^2 * Education Level 2	-0.0003	0.001	-0.56	0.57
Age^2 * Education Level 3	0.001	0.001	2.06	0.04*
Age^2 * Education Level 4	0.001	0.001	2.13	0.03*
Sex * Education Level 2	-0.28	0.23	-1.21	0.23
Sex * Education Level 3	-0.09	0.23	-0.41	0.68
Sex * Education Level 4	-0.57	0.22	-2.56	0.01*

$p < 0.01$ ** , $p < 0 .05$ *
